# An overview of the integration and development trends between arts and psychotherapy

**DOI:** 10.3389/fpsyg.2025.1617700

**Published:** 2025-07-30

**Authors:** Yuxing Xie, Xianhua Sun

**Affiliations:** College of Art and Design, Nanjing Forestry University, Nanjing, China

**Keywords:** cultural interconnections, art therapy, integration of the arts, health psychology, bibliometric analysis, CiteSpace

## Abstract

**Introduction:**

In the field of mental health, the integration of art and psychotherapy has become an important development direction. Its core mechanism lies in promoting individuals to achieve emotional release, cognitive reconstruction, and social function recovery through the creative expression of art forms, which has given rise to the important practical field of art therapy. This study aims to systematically sort out the research status and development trends in the field of art therapy from 2005 to 2024 through knowledge mapping analysis, providing references for theoretical innovation and practice standardization in this field.

**Methods:**

Based on the Web of Science core collection data sources, the CiteSpace bibliometric tool was used for knowledge mapping analysis of art therapy-related research. By searching keywords such as “Art Healing” and “Art Therapy,” 1799 valid articles were finally included after strict screening. The analysis was carried out from multiple dimensions: basic information (changes in the number of publications, journal distribution, regional and author-institution distribution) and research content (keyword co-occurrence, cluster mining, and timeline deduction).

**Results:**

Art therapy research from 2005 to 2024 showed a phased growth characteristic, successively going through a germination period, a stable period, and a rapid development period. In terms of geographical distribution, Europe and the United States, represented by the United States and the United Kingdom, contributed 63% of the core literature. Research hotspots gradually shifted from early basic intervention research to the exploration of technological integration and ethical issues. It was also found that this field is highly related to multiple disciplines such as psychology and medicine, but there are limitations such as imbalanced development of cross-cultural theories.

**Discussion:**

Although significant progress has been made in the theory and practice of art therapy, there are still structural problems such as uneven development of cross-cultural theories, insufficient connection between technical applications and practice guidelines, and inadequate research coverage on special groups. Future research can focus on the in-depth integration of theory and practice, cross-cultural theoretical integration, construction of multi-dimensional evaluation systems, and application of virtual reality technology, providing new paths to promote theoretical innovation and practice standardization in art therapy.

## 1 Introduction

Globally, mental health is a growing problem, and the population is aging; two societal trends have led to widespread concern about mental health (Wilhelm et al., 2004; Chand and Tung, 2014). Mental health problems not only affect the quality of life and work efficiency of individuals but also pose a particular obstacle to the stability and development of society. The increasing aging of the population has brought to the fore the special needs of the elderly, especially in the areas of psychological care and cognitive maintenance. Thus, the novel approach of combining art and psychotherapy has received international attention.

Art Therapy (AT) is a unique and fruitful psychotherapeutic approach. It integrates art and psychological theories to provide an effective way for people to explore their inner world, promote emotional expression, and achieve spiritual growth (Pelowski et al., 2016). Art therapy is a form of psychotherapy that uses artistic media (e.g., painting, sculpture, music, etc.) as an intervention to help individuals deal with emotional and psychological issues through creative expression. Different art forms embody distinct aesthetic characteristics—such as the visual symbolism of painting, the tactile expressiveness of sculpture, and the rhythmic dynamics of music—each of which shapes a unique emotional environment for patients. Existing research demonstrates that such environment-specific artistic contexts significantly influence psychological outcomes. For example, music therapy has been found to reduce depressive symptoms and anxiety levels in patients with depression (Aalbers et al., 2017). The arts enable humans to realize the fundamental dynamics of creation and the empowerment of possibilities. Through creative expression, patients regain wholeness, individually and as part of a larger world (Ettun et al., 2014). As mental health professionals discover that different art therapies, such as theatre, art, music, and dance, have great potential to reach and help people, more and more clients can embrace these independent art therapies (Jones, 2020). Engaging in the arts, whether as a spectator of others’ creative endeavors or as an initiator for one’s own, can elevate one’s mood and other mental states and significantly influence vital physiological parameters (Staricoff et al., 2003; Davies et al., 2015).

Scholarly interpretations of AT reflect evolving disciplinary intersections. George Bolian’s Art Interpretation and Art Therapy represents the results of the Fifth International Symposium on Expressive Psychopathology, held in Los Angeles in 1968, which was rich in film, psychodrama, and other forms of expression, and which, although it did not go into a deeper understanding of art therapy, reflected the attention it was already receiving at the time and its interdisciplinary character (Fischer and Jakab, 1969). While the work lacked deep theoretical rigor, it highlighted AT’s interdisciplinary potential. Building on this early foundation, Linney Wix and Josie Abbenante, in Archetypal Art Therapy, proposed three core tenets of archetypal art therapy: focusing on the content and structure of images, processing images imaginatively rather than symbolically, and the therapist’s engagement with the image’s metaphorical language. They argued that guiding individuals into a state of fantasy surpasses diagnostic explanations in therapeutic value. Their emphasis on metaphorical understanding of images and encouraging exploratory practices slow down the expectation for answers, thus creating more therapeutic space (Abbenante and Wix, 2015). A stance that prioritizes experiential exploration over clinical categorization.

Subsequently, scholars have expanded the scope of art therapy from different perspectives. Katie Fuller highlighted the role of art in trauma healing and the application of affect theory (A body of theory that explores the production and transmission of emotions and their sociocultural effects, emphasizing the impact of emotions as non-cognitive experiences on individual behavior and group interaction). She posited that art can be a powerful healing tool by confronting negative emotions within the body, with the emotional dimension being the linchpin of the therapeutic process (Fuller, 2020). By comparison, Patrice Rancour and Terry Barrett’s perspective emphasizes the role of artistic interpretation in clinical interventions, where observing artworks and interacting with others can help individuals express emotions, improve cognitive skills, and develop the ability to cope with health challenges (Rancour and Barrett, 2011). However, Aimie Purser’s research provides theoretical support for dance as a healing art. By combining dance with philosophical theories, she emphasizes the role of dance in connecting self, world, and others and its potential as a positive humanizing experience (Purser, 2019). These theories reveal a tension: while some prioritize emotional embodiment (Fuller), others emphasize cognitive reconstruction (Rancour and Barrett) or existential connection (Purser).

The historical development of art therapy and its origins can be traced back to *Bildnerei der Geisteskranken* by Hans Prinzhorn, a German art historian and psychiatric researcher (Prinzhorn, 1923). This seminal work bridged the fields of psychiatry and art, sparking subsequent explorations. Inspired by Prinzhorn’s findings, artists and critics developed theories such as ‘primitive art’ and ‘outsider art’ (Refers to art forms created by creators without formal artistic training (e.g., people with mental illness, folk craftsmen, etc.), emphasizing spontaneity and non-traditionalism, and often featuring strong personal expression), which, as non-traditional art practices, initially broadened the scholarly perception of the therapeutic potential of art through the spontaneity and non-professionalism that characterized their expression. In light of such practices, Margaret Naumburg formally introduced the concept of ‘art therapy’ around 1940. She systematically applied it to clinical treatment in psychiatric hospitals, establishing an early paradigm for integrating art therapy and psychology. With the maturity of the theory and changes in social needs, the application of art therapy has gradually extended from clinical psychology to a broader range of non-medical scenarios, such as education and community counseling, forming a diversified system of practice (Naumburg, 1955). Notably, this historical progression reveals a shift from viewing art as a diagnostic tool (Prinzhorn) to a therapeutic intervention (Naumburg), yet early theories lacked standardized frameworks for clinical application. These evolutionary steps collectively illustrate how art therapy has transformed from a niche concept to an interdisciplinary field, thanks to the cumulative efforts of these scholars and practitioners.

Numerous academics in related domains have written in-depth papers about subjects like neuroaesthetics (A cross-discipline combining neuroscience and aesthetics, researching the neural mechanisms and cognitive responses in the process of art appreciation and creation using brain-imaging technology.), the creative arts, mental health, complementary therapies, and rehabilitation, yet the field of AT still has a lot of untapped research potential. Current studies have demonstrated a clear gap between theoretical progress and practical implementation, frequently resulting in poor results and ethical problems (Moon and Nolan, 2019). For instance, the interdisciplinary nature of AT—combining psychology, art, and neuroscience—challenges standardizing intervention protocols. This study aims to synthesize and arrange previous research to create a strong theoretical framework for AT development, convert theoretical understanding into workable solutions that complement AT organizations, and eventually encourage sustained AT development through improved organizational policies (Bosgraaf et al., 2020a; Gerber et al., 2023).

The development of global art therapy professional organizations is characterized by geographical differences and conceptual integration, with associations in different countries and regions playing a unique role in promoting the standardization and localization of art therapy. The American Art Therapy Association (AATA), established in 1969, took the lead in promoting the development and standardization of art therapy, while the American Dance Therapy Association (ADTA), the North American Theatre During the same period, the American Dance Therapy Association (ADTA), the North American Drama Therapy Association (NADTA) and other related organizations have also emerged one after another, jointly constructing a diversified art therapy system (Stoll, 2005; Junge, 2010). Among them, the American Art Therapy Association is significant in the history of art therapy, and the Wisconsin Art Therapy Association (WATA), led by Wayne Ramirez, has led to an inclusive definition that guarantees the equal development of the fields of psychiatry, education, and community practice (Potash, 2019). In Europe, the founding of the British Association of Art Therapists (BAAT) and the British Association for Drama Therapy (BADth) pushed the professionalization of art therapy to a new height (Burton, 2009; Karkou et al., 2011); in Canada, after the establishment of the Canadian Association of Art Therapists (CATA) in 1977, BCATR and OATA were born in 1978 to improve the regional art therapy system. The Australian and New Zealand Association of Art Therapists (ANZATA), relying on multicultural backgrounds, integrating European and American concepts, and relying on close contact with Southeast Asian representatives, such as Singapore, continue to explore and innovate in cross-cultural art therapy practice. In addition, countries and regions such as Hong Kong, Singapore, South Korea, and Japan have also set up local art therapy professional organizations, focusing on local training, research, education, and industry development. Together, these practices paint a rich picture of the development of art therapy worldwide, as detailed in Table 1.

**Table 1:** National AT-related organizations.

Country/area	Related organizations	Year of establishment	Category
USA	American Art Therapy Association, AATA	1969	Art
	American Dance Therapy Association, ADTA	1966	Dance
	North American Drama Therapy Association, NADTA	1979	Drama
	American Music Therapy Association, AMTA	1998	Music
	Wisconsin Art Therapy Association, WATA	1969	Art
England	British Association of Art Therapists, BAAT	1940	Art
	British Association of Drama Therapists, BADth	1977	Drama
Canadian	Canadian Association of Art Therapists	1977	Art
	Canadian Art Therapy Association, CATA	1977	Art
	British Columbia Art Therapy Association, BCATR	1978	Art
	Ontario Art Therapy Association, OATA	1978	Art
Australia New Zealand	ANZATA	1987	Art
Hong Kong, China	Expressive Arts Therapy Association of Hong Kong, EATAHK	2012	Art
Singaporean	Singapore Music Therapy Association	2007	Music
South Korea	Korean Dance Association	1961	Dance
Japan	Japan Dance Therapy Association	1992	Dance
China-Taiwan	Taiwan Dance Therapy Association	2010	Dance

AT has become an important part of future social development, and many countries around the world have set up professional associations to actively promote AT training, research, and education in their local communities, helping individuals to achieve psychological recovery and growth and promoting the improvement of the social mental health system (Gilroy, 2006; Kapitan, 2011; Case and Dalley, 2014). This study aims to explore the theory and practice of AT in a multi-dimensional and systematic way. First, we systematically sort out the theoretical evolution of AT, track the development of industry associations in major countries and regions around the world, and construct a complete theoretical development map; second, we focus on the research progress and disciplinary distribution of AT, analyze the research hotspots and trends, and explore the potential direction of disciplinary cross-fertilization; and third, based on the results of the existing research, we develop a methodological approach, data support, and theoretical framework. Thirdly, based on the existing research results, we will critically reflect on the methodology, data support, and theoretical framework and prospectively propose a path for AT research to deepen in the future to guide the field’s theoretical innovation and practical development.

Building a thorough assessment system that can be quickly adjusted and tuned to the AT culture is crucial to closing the gap between theory and practice and expanding the therapeutic applications of the various arts. Thus, this study determines AT’s key components and keywords by identifying and analyzing AT trends. Examine current hot issues, research trends, and possible avenues for future expansion, optimization, and research using CiteSpace (Zhou and Wang, 2019; Li et al., 2021). The analysis aims to offer direction and heipful information for AT research (Chen, 2006; Liang et al., 2023; Xing et al., 2024). In addition to the historical evolution analysis, the WoS database indicates the future research path for AT. This offers a fresh viewpoint on the path and direction of AT research and acts as a theoretical guide for the superior advancement of AT in various countries. It is important to note that this study examines the AT hotspots differently than other research; it builds on earlier findings and poses specific queries to investigate novel approaches for future investigations. Specifically, this study focuses on the following key issues: combining the research progress in the field of art therapy, outlining the research ecology in terms of the number of publications, major journals, core countries or regions, authors’ collaborative networks, and institutional distribution; secondly, analyzing the characteristics of the distribution of disciplines in the field, and analyzing the patterns of cooperation between disciplinary categories and the academic influence of highly cited papers; thirdly, mining the research hotspots and thirdly, the research hotspots and development trends of art therapy will be explored, and keyword co-occurrence analysis, cluster mining, and timeline projection will be used to reveal the evolution and future direction of the discipline, which will provide the basis for promoting the deepening of theories and expanding the practice of art therapy.

The structure of this paper is also corresponding; firstly, section 1 outlines the theoretical evolution of AT, systematically combs its core theoretical iterative process, and conducts a panoramic scan of the practical progress of industry associations in major countries and regions around the world, and section 2 details the data sources and methodological framework, clarifying the scope of databases for literature searches, the criteria for data cleansing, and the research methodology. Subsequently, Section III provides a detailed analysis based on the research progress, research areas, research hotspots, and research trends of AT. The discussion in Section 4 builds a critical and forward-looking discussion system by comprehensively interpreting the empirical results across dimensions, systematically reflecting on the research from the data limitations/methodological applicability/theoretical framework level, and proposing future challenges and development paths of AT research in the light of the academic frontiers. The final part of the study refines the study’s core findings, formulates strategic recommendations for the development and practical application of AT theory, and points out the direction for further research.

## 2 Materials and methods

### 2.1 Data sources

Choosing reference journal articles from reputable sources was the first step in building a consistent database for analysis in this study. The database used in the study is the WoS platform. Worldwide, the WoS core database is acknowledged as authoritative (Hou et al., 2018). The database contains over 20,000 globally recognized, highly influential academic publications and conference papers in addition to a range of other resources, covering natural sciences, arts, humanities, and many other fields with an authoritative and scientific nature (Mingers and Leydesdorff, 2015).

**Figure 1:**
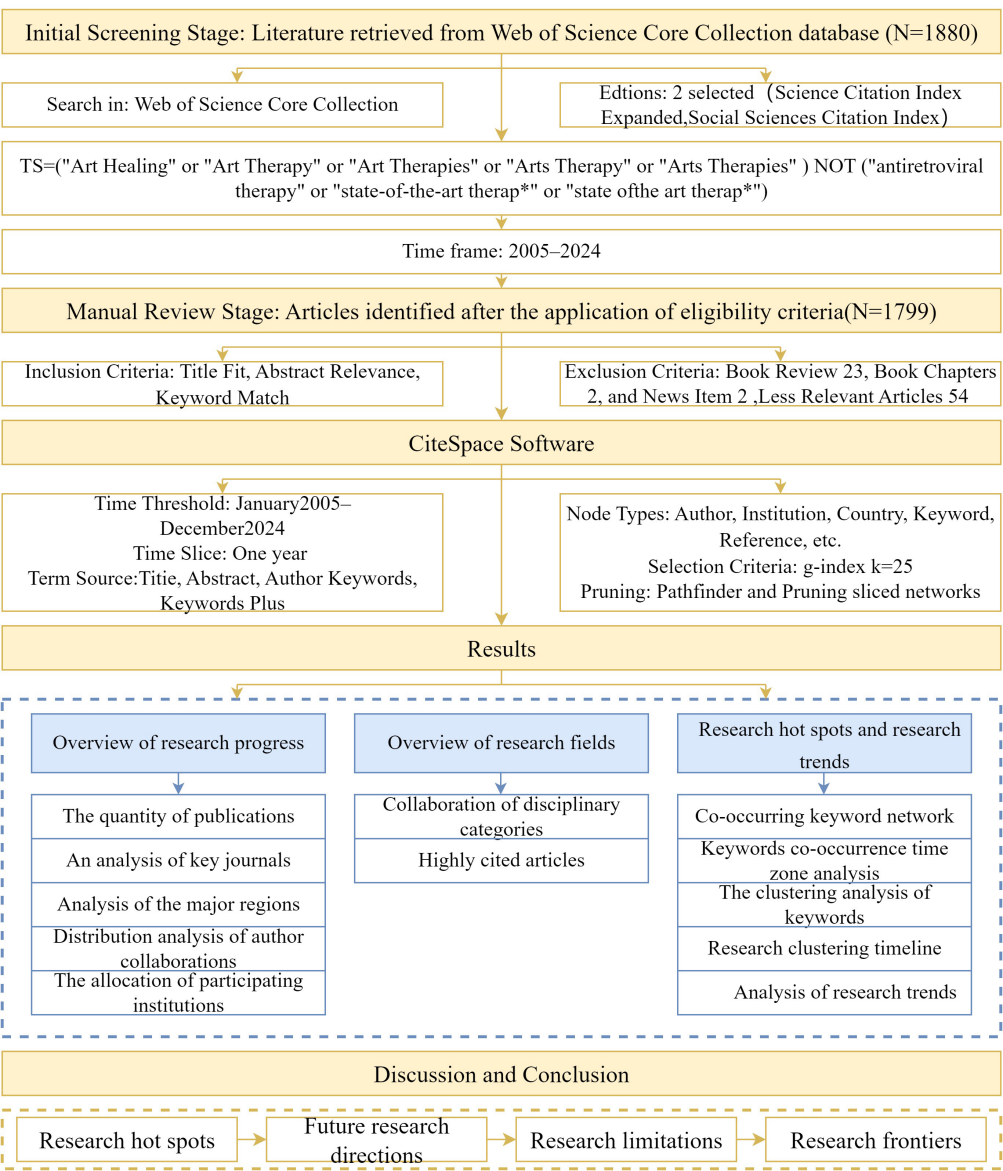
AT study design outline.

The second step is to choose the right keywords (Taherdoost, 2016). The topic of this study is research on the integration and trends of art and psychotherapy. In the advanced search, to ensure the study’s accuracy, we chose Web of Science Core Collection, Editions: 2 selected (Science Citation Index Expanded, Social Sciences Citation Index). TS = (“Art Healing” or “Art Therapy” or “Art Therapies” or “Arts Therapy” or “Arts Therapies”) NOT (“antiretroviral therapy” or “state-of-the-art therap*” or “state of the art therap*”). The search time frame was set to 2005 to 2024 when the number of articles was 1880. Excluding other types of literature, such as Book Review 23, Book Chapters 2, and News Item 2, the total number of articles was 1853. By reviewing and organizing, 54 articles with low relevance (e.g., Antiretroviral Therapy, ART, and Assisted Reproductive Technology, ART) were excluded, resulting in 1799 valid articles. The present review was conducted according to the PRISMA guidelines; see Figure 1 for the detailed screening flowchart (Rethlefsen et al., 2021).

### 2.2 Research methodology

CiteSpace is used as an analysis tool in this study (Ding and Yang, 2022). CiteSpace is a bibliometric analysis tool developed by Professor Chaomei Chen, which is mainly used for the visual analysis of scientific literature, revealing the research hotspots and development trends in the subject areas by drawing co-citation networks and keyword clustering maps. It is a Java-based application for information analysis and visualization that illustrates scientific knowledge hierarchies through data visualization (Song and Zhao, 2015). It is helpful for pinpointing subjects at the forefront of widely accepted research. Researchers can concentrate on more critical analytical problems, creative thinking, and abstract thinking by using computer algorithms and interactive visualizations instead of conventional bibliometric techniques. A “bibliometric map” or “knowledge map” is the resultant graph.

In this study, the literature on art therapy over the last decade was visualized and analyzed with the help of CiteSpace 6.4. R1 to identify core research themes and theoretical evolutionary paths in the field (Rodriguez Novo et al., 2021). After exporting 1799 bibliographic records into a dataset containing plain text content and full citation references, they were imported into CiteSpace 6.4. R1 visual analysis software for computational processing. The study set the time slice as the one-year unit, and the rest of the parameters were configured by the software default. The system extracted the core data such as keywords, authors’ information, and names of the institutions in the literature to start the analysis.

In order to explore the progress of research in the field of art therapy, this study conducted a multidimensional visual analysis of 1799 articles based on scientometric methods. In the dimension of research progress overview, we analyzed the distribution of publications, identified key journals, and presented the distribution patterns of major research regions, author collaboration networks, and participating institutions; in the dimension of research field overview, we explored the collaboration patterns of disciplinary categories and explored the highly cited literature. In terms of research hotspots and trends, with the help of keyword co-occurrence network, keyword co-occurrence time and time zone analysis, keyword clustering, research clustering timeline, and other methods, the hot keywords of each period are presented visually. The field’s current research status and development trend from 2005 to 2024 are dynamically analyzed to provide an all-rounded and systematic insight into the evolution path of the research in art therapy. The AT study design is shown in Figure 1.

## 3 Results

### 3.1 Overview of research progress

#### 3.1.1 The quantity of publications

**Figure 2:**
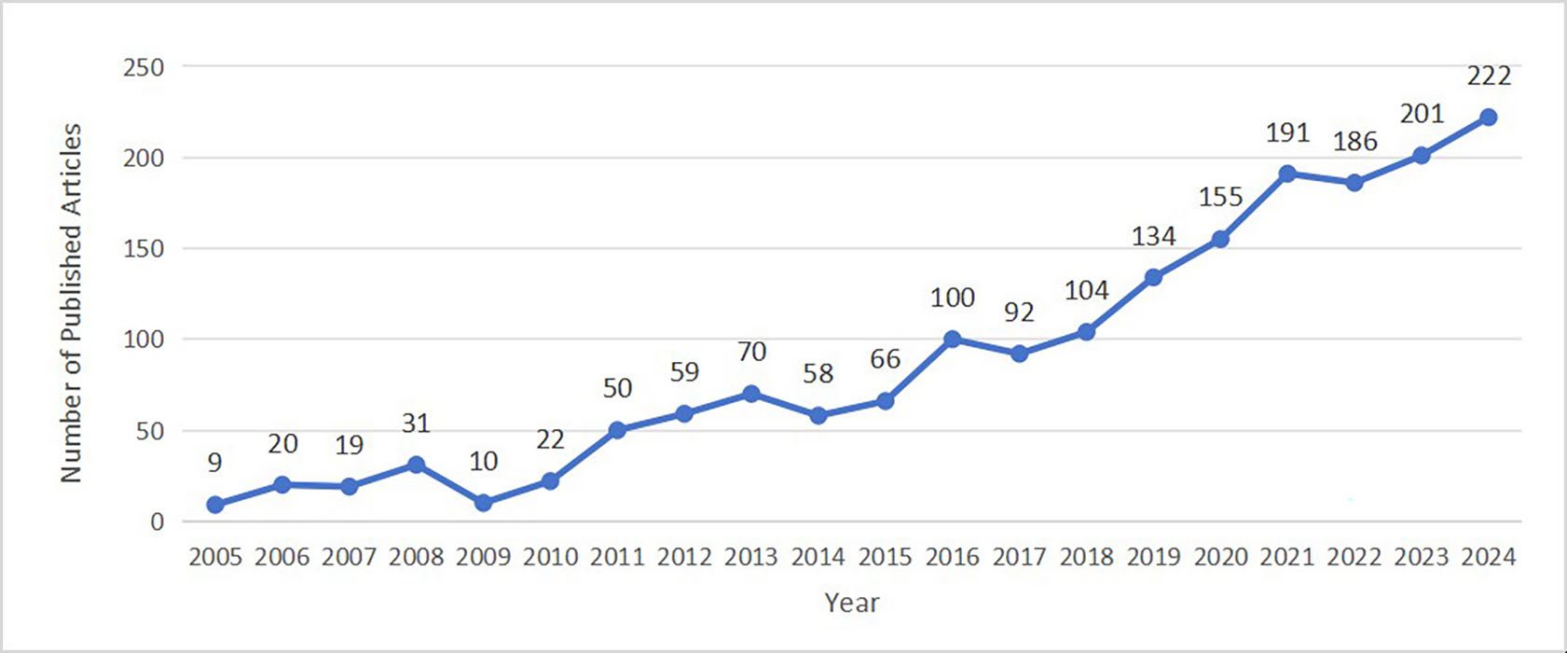
Data on AT research paper production from 2005 to 2024.

The annual change in the number of publications is an important quantitative measure of the development of the discipline. Based on the core database of WoS, this study systematically counts the annual output of literature in AT from 2005 to 2024. It reveals the development of the discipline through the trend analysis of the time series data. It is found that academic research in this field during the period of 2005–2024 presents a stage-by-stage evolutionary feature, which can be specifically divided into three developmental stages, namely, the budding start period (2005–2014), the steady growth period (2015–2019) and the rapid rise period (2020–2024), as shown in Figure 2.

##### 3.1.1.1 Unstable or fluctuating growth (2005–2014)

The annual number of articles published during this time was comparatively low. It varied greatly, with only nine articles published in 2005, compared to 58 in 2014, and the overall number of articles remaining largely below 70. The overall number is under 50 articles, showing an unstable state with no obvious increasing or decreasing trend. The field is still in its early stages of development, and research findings are not steady.

During the start-up period 2005–2014, global art therapy research output was erratic and low in volume, influenced by three main factors. First, the theoretical system has not yet been perfected. Although North America and Europe initially constructed art therapy theories in the 20th century, there is insufficient international consensus on the theoretical framework, which makes it difficult to form a synergy in cross-regional research. Secondly, there are significant cross-disciplinary barriers. As a cross-field of psychology and art, art therapy lacks an independent disciplinary status in the international academic system, and it is not easy to integrate resources. Thirdly, there are social cognitive limitations. At that time, the international community’s attention to mental health treatment was focused on medication and talking therapies, and the non-traditional intervention form of art therapy was not widely recognized. This stage of exploration laid the foundation for subsequent development, and some of the pioneering studies (such as the intervention experiments with traumatized populations carried out by the American Art Therapy Association) provided an early experience for an international standardized research paradigm, which pushed art therapy gradually from a fringe discipline to a mainstream academic perspective.

##### 3.1.1.2 Slow growth or stable development (2015–2019)

There has been a more consistent rise in articles published since 2015. From 66 articles in 2015 to 134 in Zhou and Wang, 2019, the annual increase in number, although not particularly significant, has been on an upward trend with low fluctuations. This stage denotes a slow but steady rise in the quantity of research findings, a gradual maturation of the pertinent research methodologies, a rise in the number of researchers, and a growing interest in the area.

The steady growth of art therapy research from 2015 to 2019 is due to a positive shift in the international environment. Firstly, the global mental health crisis has been highlighted, with the World Health Organisation (WHO) reporting in 2017 that more than 300 million people suffer from anxiety and depression disorders, prompting countries to begin exploring diversified treatments, and art therapy has gained attention for its combination of humanistic care and intervention effects. Second, strengthening international academic communities, such as the International Art Therapy Association (EATA), has promoted the sharing and integration of cross-cultural research findings through global conferences and the publication of industry white papers. Furthermore, the spread of technology has provided a boost, and the development of online academic platforms has broken down geographical constraints, making it easier for researchers in Asia, Africa, and Latin America to participate in international collaborations. Steady progress in this research phase has led to art therapy taking its place in the international mental health delivery system. WHO is inclusion of art therapy in the Mental Health Gap Action Plan in 2018 has accelerated the internationalization of the discipline by promoting pilots of its application in low- and middle-income countries.

##### 3.1.1.3 Rapid growth or development (2020–2024)

The growth in the number of published articles accelerated significantly after 2020. There were 155 articles in 2020, reaching 222 in 2024, an average of 190 articles per year in just a few years. This stage shows that the field has entered a period of rapid development, attracting many researchers due to the emergence of new research hotspots, technological breakthroughs, and more financial and policy support, leading to the rapid output and publication of research results.

The explosive growth of art therapy research after 2020 is closely linked to multiple changes in the international community. COVID-19 has triggered a global psychological crisis, with UNESCO data showing a 40% surge in demand for mental health counseling globally between 2020 and 2022, and tele-art therapy becoming an important intervention due to its lack of physical space constraints. At the same time, interdisciplinary technological breakthroughs provide new momentum, and the application of neuroscience and artificial intelligence technologies (such as fMRI to monitor the activation of brain regions during art creation) has given rise to cutting-edge directions such as “neuro-art therapy.” In addition, international policy support has increased, with the European Union’s Horizon 2020 program and the US National Institutes of Health (NIH) Special Fund including art therapy in their funding. The period of rapid growth has significantly enhanced the international discourse of art therapy, and the establishment of the International Art Therapy Research Network (IATRN) in 2023 signaled the formation of a global collaborative ecology for the discipline. However, the discipline also faces new challenges, such as theoretical fragmentation and lack of cultural appropriateness, and there is an urgent need to establish a unified international research standard.

#### 3.1.2 An analysis of key journals

Looking at the major journals to see which ones have examined AT in greater detail can play a crucial part in deciding the path of future study. This will help future researchers by giving them helpful research directions.

**Figure 3:**
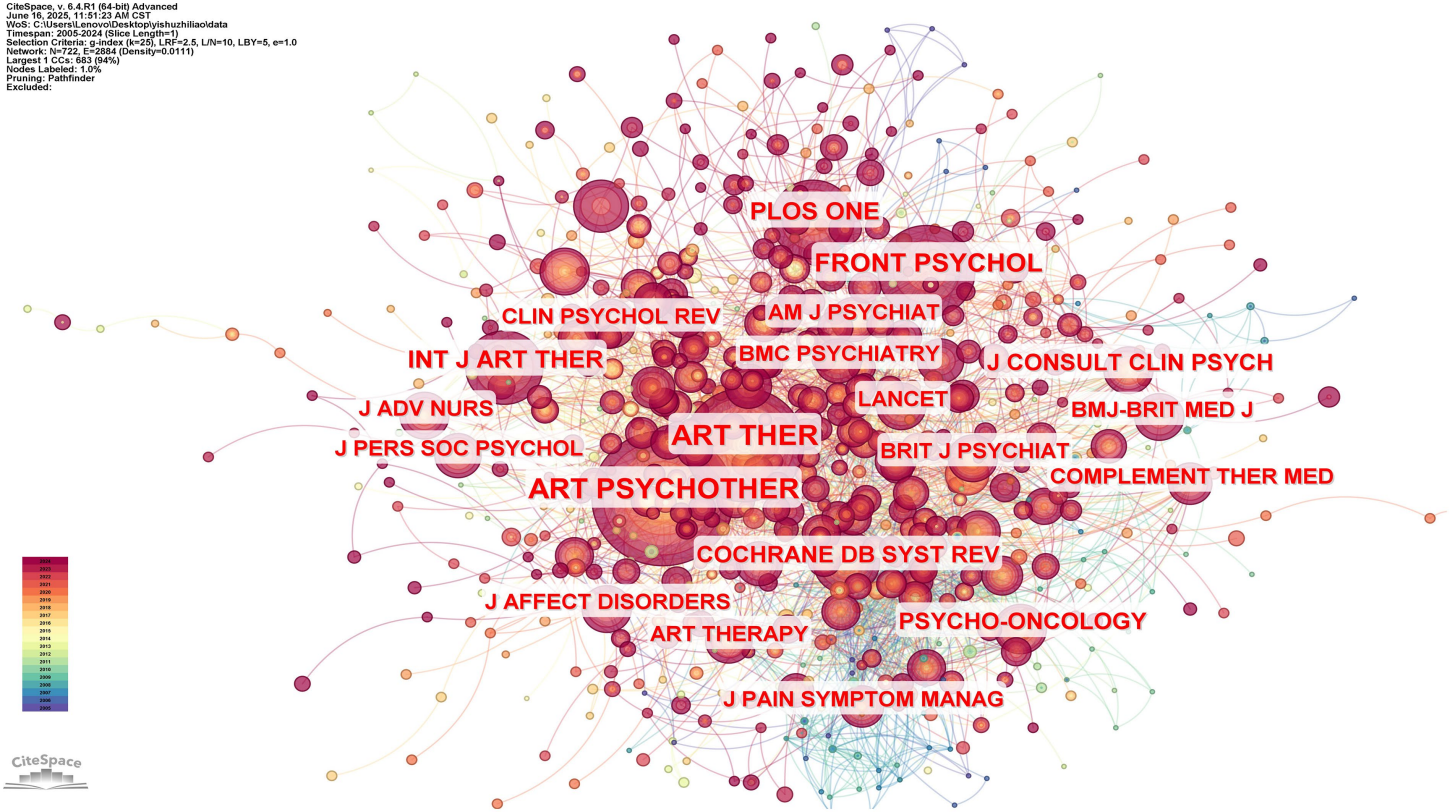
Knowledge mapping of AT collaborative journal publishing research, 2005–2024.

The co-cited journals map was created by setting the node type to “cited journals” and the time slice to one year. According to the analysis, the journal distribution has 722 nodes overall, with a grid density of 0.0111 and a connection value of 2,884. Table 2 shows that the top five journals with the highest number of publications are ART PSYCHOTHER (867), ART THER (666), FRONT PSYCHOL (402), PLOS ONE (296), and INT J ART THER (266). The journals show a specific hierarchical distribution. This distribution indicates that the research results in art therapy are not concentrated in individual journals but are reflected in multiple journals. Journals with different frequencies of publication together build an ecosystem for the publication of art therapy research results, which meets the publication needs of scholars at different levels and in different research directions; according to the corresponding publishers of the journals, it can be seen that the journals are issued by well-known academic publishers such as Elsevier, Taylor & Francis, John Wiley, and Sons, etc. In particular, Elsevier and John Wiley and Sons Ltd. appear twice in the table, respectively, and with their global influence, they can put the published art therapy, they can promote their published art therapy journals all over the world, which facilitates international exchanges in the field of art therapy. Most of these major research journals are from Europe and the United States, such as the Netherlands, the United Kingdom, and the United States. This reflects the dominance of research in art therapy in Europe and the United States. The knowledge graph that was exported using CiteSpace is seen in Figure 3. We can observe the relationships between the authors of each publication and determine which journals publish more articles by looking at Figure 3. Based on original research and the journal’s disciplinary approach, conclusions are made. The study’s findings demonstrate that the advancement of AT may directly affect the disciplines of sociology, psychology, medicine, and the arts.

**Table 2:** Major research journals in AT 2005–2024.

No.	Freq	Centrality	Cited journals	Publishers	Country
1	867	0.01	ART PSYCHOTHER	Elsevier	HOLLAND
2	666	0.01	ART THER	Taylor & Francis	ENGLAND
3	402	0.01	FRONT PSYCHOL	Frontiers Media S. A.	SWITZERLAND
4	296	0.01	PLOS ONE	Public Library of Science	UNITED STATES
5	266	0.01	INT J ART THER	Taylor & Francis Ltd	ENGLAND
6	236	0.03	COCHRANE DB SYST REV	John Wiley and Sons Ltd	ENGLAND
7	214	0.01	PSYCHO-ONCOLOGY	John Wiley and Sons Ltd	ENGLAND
8	179	0.03	J CONSULT CLIN PSYCH	APA	USA
9	175	0.04	CLIN PSYCHOL REV	Elsevier	HOLLAND
10	171	0.02	BMJ-BRIT MED J	BMJ Publishing Group	ENGLAND

#### 3.1.3 Analysis of the major regions

Important details about the primary nations where AT is researched can be found by analyzing the distribution of regional cooperation in publications. This study also aids in identifying potential directions for art therapy research. By analyzing the knowledge map, it is possible to see which countries are more innovative and which have carried out earlier research in art therapy, thus offering researchers in nations whose research has lagged behind practical implications.

Our analysis of the regional cooperation distribution of publications in the AT area using CiteSpace revealed that the United States, China, Israel, and the United Kingdom had the most regional collaborations. In contrast, the other six countries had very few papers. Table 3 illustrates that the USA, ENGLAND, AUSTRALIA, etc., began their research earlier. However, nodes with centrality values higher than 0.1 are considered crucial nodes when looking at centrality, and these nodes are generally considered significant causes that cause changes in the studied area (Wenjuan, 2012). The literature centrality values for the USA (0.34), CHINA (0.20), ISRAEL (0.10), ENGLAND (0.25), GERMANY (0.26), AUSTRALIA (0.36), ITALY (0.13) are all more than 0.1, as Table 3 demonstrates. These seven nations are pretty innovative and significantly impact art therapy research. However, there is a significant geographical imbalance in current research, with the United States, the United Kingdom, and Israel contributing 63% of the core literature. In comparison, East Asian countries such as China and Japan account for only 9.8% of the research; most of it focuses on clinical interventions and lacks a focus on indigenous theories.

**Table 3:** The amount of documents pertaining to AT research and the list of contributing nations, 2005–2024.

No.	Freq	Centrality	Year	Country
1	429	0.34	2005	USA
2	177	0.20	2010	PEOPLES R CHINA
3	162	0.10	2007	ISRAEL
4	162	0.25	2005	ENGLAND
5	130	0.26	2007	GERMANY
6	101	0.36	2005	AUSTRALIA
7	85	0.00	2011	SOUTH KOREA
8	83	0.07	2008	NETHERLANDS
9	74	0.13	2006	ITALY
10	72	0.09	2006	CANADA

**Figure 4:**
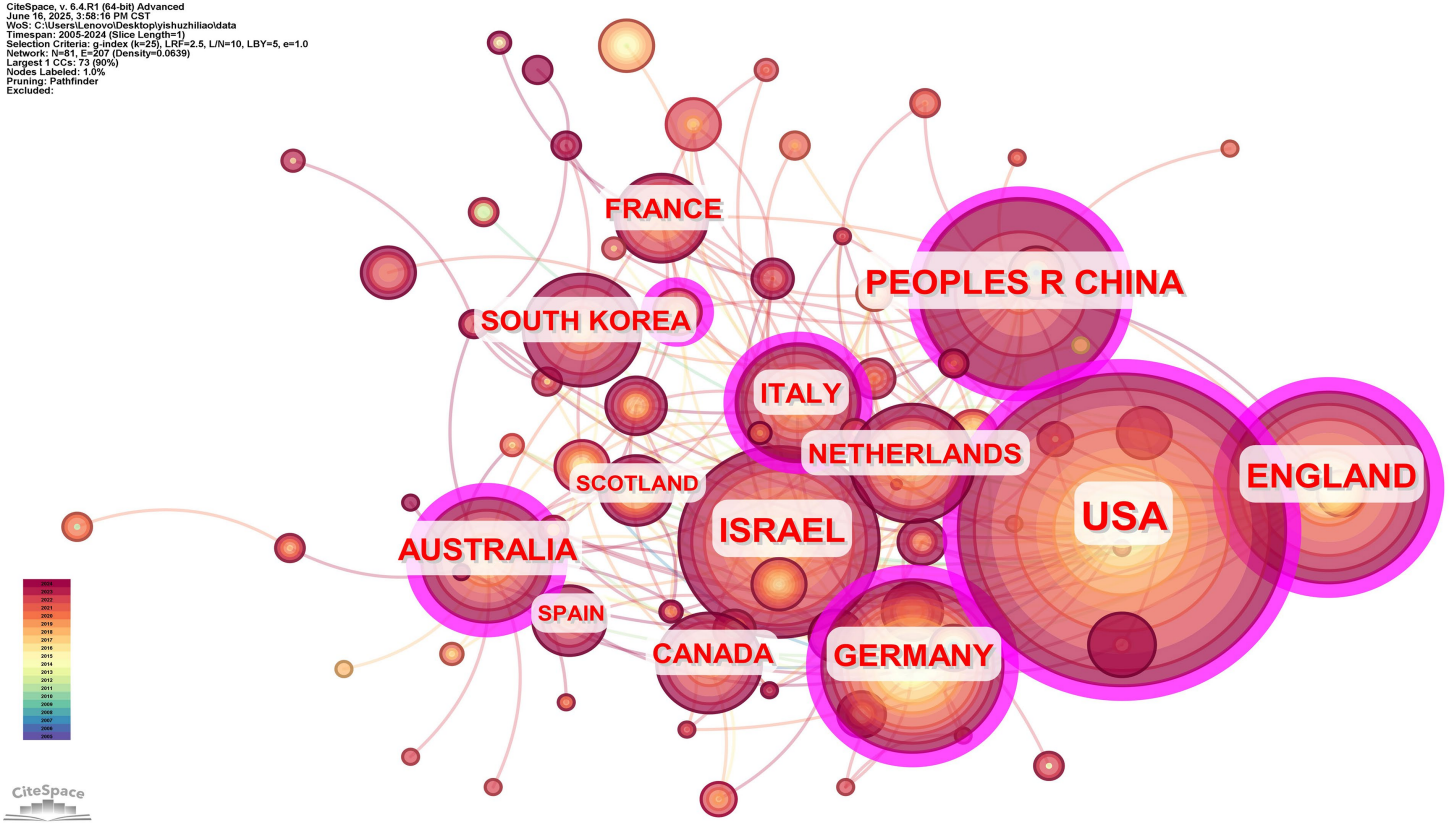
Knowledge map of nations participating in AT research collaboration, 2005–2024.

Figure 4 illustrates the graph’s 81 nodes, each with a grid density of 0.0639 and a connection value 207. The graph shows that the countries with a low level of connectivity, such as SOUTH KOREA, SPAIN, and SCOTLAND, lack cooperation with other countries. In contrast, the USA, ENGLAND, and CHINA have a high level of cooperation with other countries. The advancement of research is facilitated by international collaboration and exchange.

#### 3.1.4 Distribution analysis of author collaborations

The distributional study of author partnerships identifies the major actors in the field and establishes cross-border and collaborative links among scholars.

**Figure 5:**
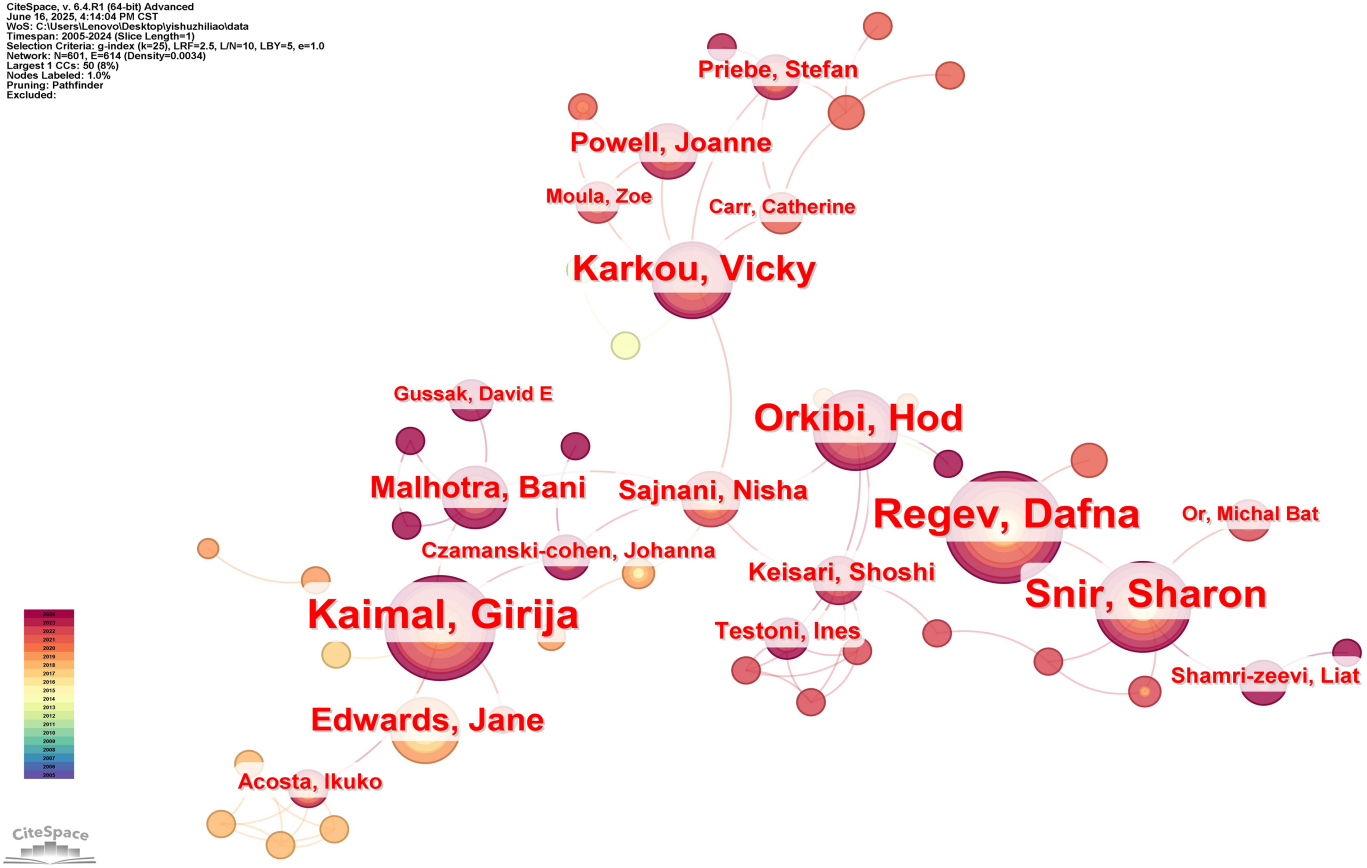
Co-authors of articles published between 2005 and 2024 are listed in an AT knowledge map.

Collaborative mapping of 1799 papers was visualized and analyzed (Figure 5), where the connecting lines between the writers show their mutual writing relationship. The size of the nodes shows how many articles each author has published, given Price’s law and the assumption that the most prolific author in a subject has max papers, m = 0.749nmax1/2. Those who have published more than m times in the discipline are considered the study’s first authors (Anna, 2021). Authors with 12 or more publications are known to be core authors (n max = 32 m ≈ 12). According to the data, the sample included seven core authors in total, with Regev, Dafna, Kaimal, Girija, Haeyen, Suzanne, Snir, Sharon, Orkibi, Hod, Karkou, Vicky, and Edwards, Jane being the lead core authors. As seen from the figure, each research team is less cooperative and more independent from the other research teams. According to the analyses, teams should communicate more cooperatively in future research processes.

According to Table 4, the two most published researchers on this topic are Kaimal Girija from the USA and Regev Dafna from Israel. Three of the top ten are from Israel, two from the USA, and one from Australia. Scholars from these three countries have made outstanding contributions to AT development. The analysis’s conclusions make it clear that AT researchers struggle to work together and that the field’s research is significantly more scattered, with little to no relationship among researchers.

**Table 4:** The top 10 authors in AT from 2005 to 2024 in terms of output.

No.	Publications	Authors	Institution	Country
1	32	Regev, Dafna	Univ Haifa	Israel
2	30	Kaimal, Girija	Drexel Univ	USA
3	26	Snir, Sharon	Tel Hai Coll	Israel
4	26	Haeyen, Suzanne	HAN Univ Appl Sci	Netherlands
5	21	Orkibi, Hod	Univ Haifa	Israel
6	18	Karkou, Vicky	Edge Hill Univ	England
7	13	Edwards, Jane	Deakin Univ	Australia
8	11	Malhotra, Bani	Drexel Univ	Philadelphia
9	9	Van lith, Theresa	La Trobe Univ	Australia
10	9	van hooren, Susan	Susan van Hooren	USA

#### 3.1.5 The allocation of participating institutions

**Figure 6:**
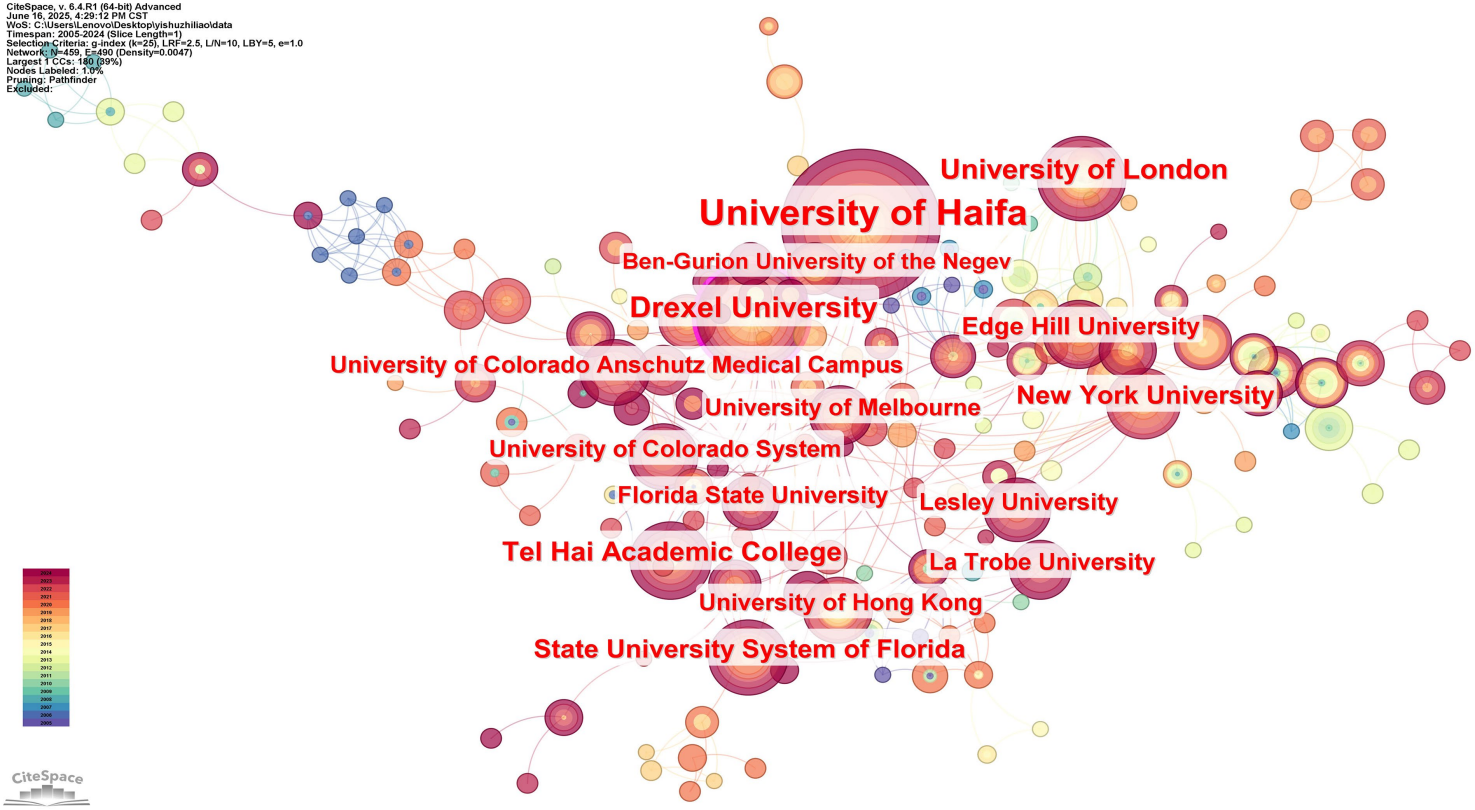
Knowledge mapping of collaborating organizations in AT research 2005–2024.

Cooperation amongst research institutes offers recognition in the sector and academic support. The AT knowledge map (Figure 6) was created with CiteSpace, with 459 nodes, 490 connections, and a grid density of 0.0047. Every node in the structure denotes the number of papers, and connections show inter-organizational cooperation; the more collaboration, the more the institution and other organizations collaborate.

Of the 10 universities that published the most papers between 2004 and 2024, according to the number of publications published, the University of Haifa (107), Drexel University (46), and the University of London (36) were the top three universities. The University of Haifa is at the top of the list. This suggests that the University of Haifa collaborates extensively with other academic institutions in AT that do in-depth research. The charts, however, show a more focused and coordinated collaboration between universities and institutions, suggesting that inter-institutional collaboration can result in more rapid and relevant research and that institutional collaboration in AT is cohesive.

Table 5 shows that six of the top 10 schools or institutions with the most publications are from the United States, which is a good indication that international cooperation is concentrated in European and American institutions and that the network of cooperation among Asian institutions is fragmented. Moreover, the number of publications is not the same as the centrality ranking, suggesting that there are indeed research institutions that publish more. However, the quality of the articles is not as high, and the impact is not as significant. There should be closer cooperation between research institutes, companies, and universities worldwide, as shown in Figure 6 and Table 5.

**Table 5:** Institutions that contribute, arranged by importance frequency and frequency.

No.	Freq	Centrality	Institution	Country
1	107	0.07	University of Haifa	Israel
2	46	0.10	Drexel University	USA
3	36	0.02	University of London	UK
4	32	0.00	Tel Hai Academic College	Israel
5	27	0.01	State University System of Florida	USA
6	24	0.03	New York University	USA
7	21	0.03	Edge Hill University	UK
8	20	0.01	University of Colorado System	USA
9	20	0.01	University of Colorado Anschutz Medical Campus	USA
10	19	0.01	Lesley University	USA

### 3.2 Overview of research fields

#### 3.2.1 Collaboration of disciplinary categories

This created a collaborative relationship graph for subject categories with the time slice set to a year and the node type set to a “category.” One hundred eighteen nodes, a grid density of 0.0406, and 280 connections make up the knowledge network, according to CiteSpace research. The magnitude of centrality indicates the degree of influence, and the 10 most influential categories are shown in Table 6. According to the table, the most influential category is PUBLIC, ENVIRONMENTAL & OCCUPATIONAL HEALTH (0.47), followed by HEALTH CARE SCIENCES & SERVICES (0.23), CLINICAL NEUROLOGY (0.22) and ONCOLOGY (0.21). According to the analysis’s results, the categories with the most significant number of publications do not have a tremendous impact, and some of these categories have low centrality, which suggests that their articles are not widely cited and that further study is necessary to improve the quality of publications. Reading publications with a high central value will help to expand the scope of future AT studies and find new avenues for investigation.

**Table 6:** List of the most important AT-related categories, 2005–2024.

No.	Freq	Centrality	WoS Categories
1	441	0.14	REHABILITATION
2	438	0.03	PSYCHOLOGY, CLINICAL
3	325	0.15	PSYCHIATRY
4	241	0.15	PSYCHOLOGY, MULTIDISCIPLINARY
5	140	0.21	ONCOLOGY
6	112	0.47	PUBLIC, ENVIRONMENTAL & OCCUPATIONAL HEALTH
7	100	0.14	NURSING
8	95	0.23	HEALTH CARE SCIENCES & SERVICES
9	94	0.09	PSYCHOLOGY
10	89	0.22	CLINICAL NEUROLOGY

**Figure 7:**
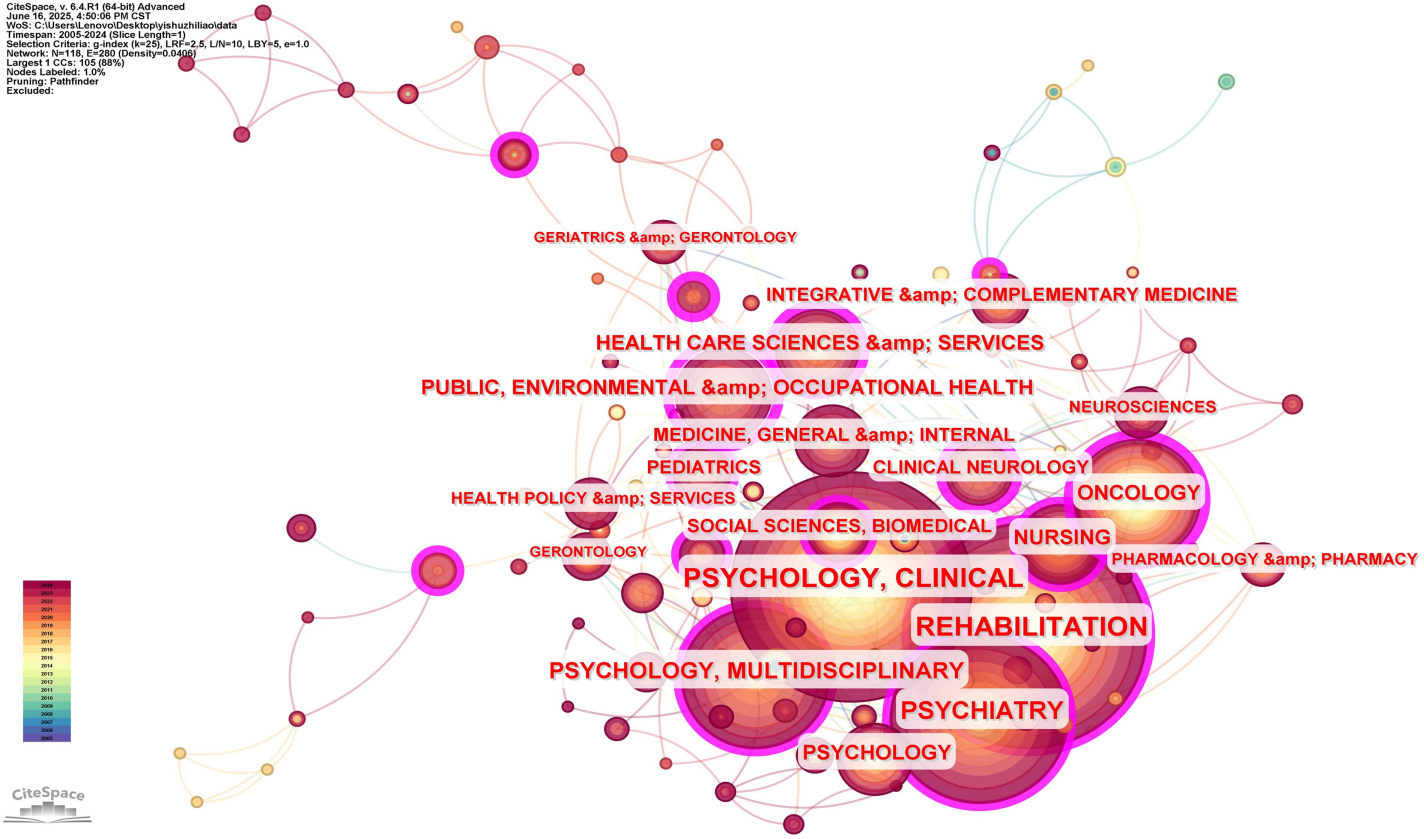
Co-occurring network of AT research topics 2005–2024.

High centrality values are indicated by the thick purple outer boxes of the nodes in Figure 7, and the connecting lines between them show relationships with other categories; the relationship is stronger when the line is thicker. The graph shows that the AT region’s primary fields include REHABILITATION, PSYCHIATRY, PSYCHOLOGY, MULTIDISCIPLINARY, ONCOLOGY, PUBLIC, ENVIRONMENTAL and occupational HEALTH, NURSING, HEALTH CARE SCIENCES and services, CLINICAL NEUROLOGY, and many others. The visualization and analysis provided by CiteSpace demonstrate how closely these fields relate to AT’s research and how interdisciplinary cooperation is crucial for future advancement.

#### 3.2.2 Highly cited articles

The articles in the WoS Core Collection that were filtered using the keywords AT and Art healing (AH) were arranged from most to least referenced. (Art healing focuses on promoting physical and mental healing through art activities, emphasizing the positive effects of art on the emotional relief and spiritual growth of individuals, and differs slightly from art therapy in its theoretical framework and practical goals.)

The article with the most citations was “From Therapeutic Factors to Mechanisms of Change in the Creative Arts Therapies: A Scoping Review” (cited 31 times) and the second most cited article was”What is the evidence on the role of the arts in improving health and well-being? A scoping review” (cited 29 times), and the third most cited is “The bodymind model: A platform for studying the mechanisms of change induced by art therapy” (cited 26 times). These are the top three most cited articles. Table 7 provides further information. These highly mentioned articles show us that there are currently three main areas of knowledge about the foundations of AT: (1) Theoretical mechanisms, such as exploring the mechanisms of change in creative arts therapy (de Witte et al., 2021), studying the principles of art therapy-induced change based on the mind–body model, and clarifying the internal logic of “how art therapy works” (Czamanski-Cohen and Weihs, 2016). (2) Empirical evidence: Answering the question of whether art healing “works” by analyzing the role of the arts in health and well-being (Fancourt and Finn, 2020) and the effectiveness of art therapy interventions for mental illness (Chiang et al., 2019). Moreover, (3) Application Practice: This program includes the application of art therapy for specific groups such as children and adolescents (Bosgraaf et al., 2020b), the direction of art therapy practice in the digital age (Zubala et al., 2021), and the exploration of the practical aspects of the associated reporting standards, in order to solve the real problem of “how to apply” art therapy.

**Table 7:** Strongest citation bursts in AT literature, 2004–2024.

No.	Title	Year	Citations	IF
1	From Therapeutic Factors to Mechanisms of Change in the Creative Arts Therapies: A Scoping Review	2021	31	2.9
2	What is the evidence on the role of the arts in improving health and well-being? A scoping review	2019	29	11.1
3	The bodymind model: A platform for studying the mechanisms of change induced by art therapy	2016	26	1.5
4	Creative art therapy for mental illness	2019	22	3.9
5	The role of emotional processing in art therapy (REPAT) for breast cancer patients	2019	22	1.5
6	The PRISMA 2020 statement: an updated guideline for reporting systematic reviews	2021	21	42.7
7	Art Therapy for Psychosocial Problems in Children and Adolescents: A Systematic Narrative Review on Art Therapeutic Means and Forms of Expression, Therapist Behavior, and Supposed Mechanisms of Change	2019	21	2.9
8	Editorial: The State of the Art in Creative Arts Therapies	2021	20	2.9
9	Art therapy for anxiety, depression, and fatigue in females with breast cancer: A systematic review	2020	20	1.5
10	Art Therapy in the Digital World: An Integrative Review of Current Practice and Future Directions	2016	20	2.9

According to an analysis of the cited articles, the AT and AH domains cover a wide range of disciplines, indicating that developing an effective AT strategy necessitates interdisciplinary cooperation and communication. There were articles with low impact factors among the highly cited ones, indicating no clear correlation between an article’s impact and the number of citations it receives. Compared to the journal in which it was published, an article with a high number of citations but a low impact factor has more influence—highly cited publications in AT show the extent and dynamics of the field’s research activities. Citations to these articles may also be used to identify the most active disciplines and research topics. We can determine present research hotspots and future trends by examining the correlation between the number of citations and the year of publication.

### 3.3 Research hot spots and research trends

#### 3.3.1 Co-occurring keyword network

Keywords serve as the primary summary of the literature and illustrate the connections between the different subjects covered in it. In contrast with co-citation analysis, keyword co-occurrence analysis can better capture the AT field’s popular content core, hotspots subject, distribution, and disciplinary structure. It can also highlight the publications’ primary content and research methodologies.

**Figure 8:**
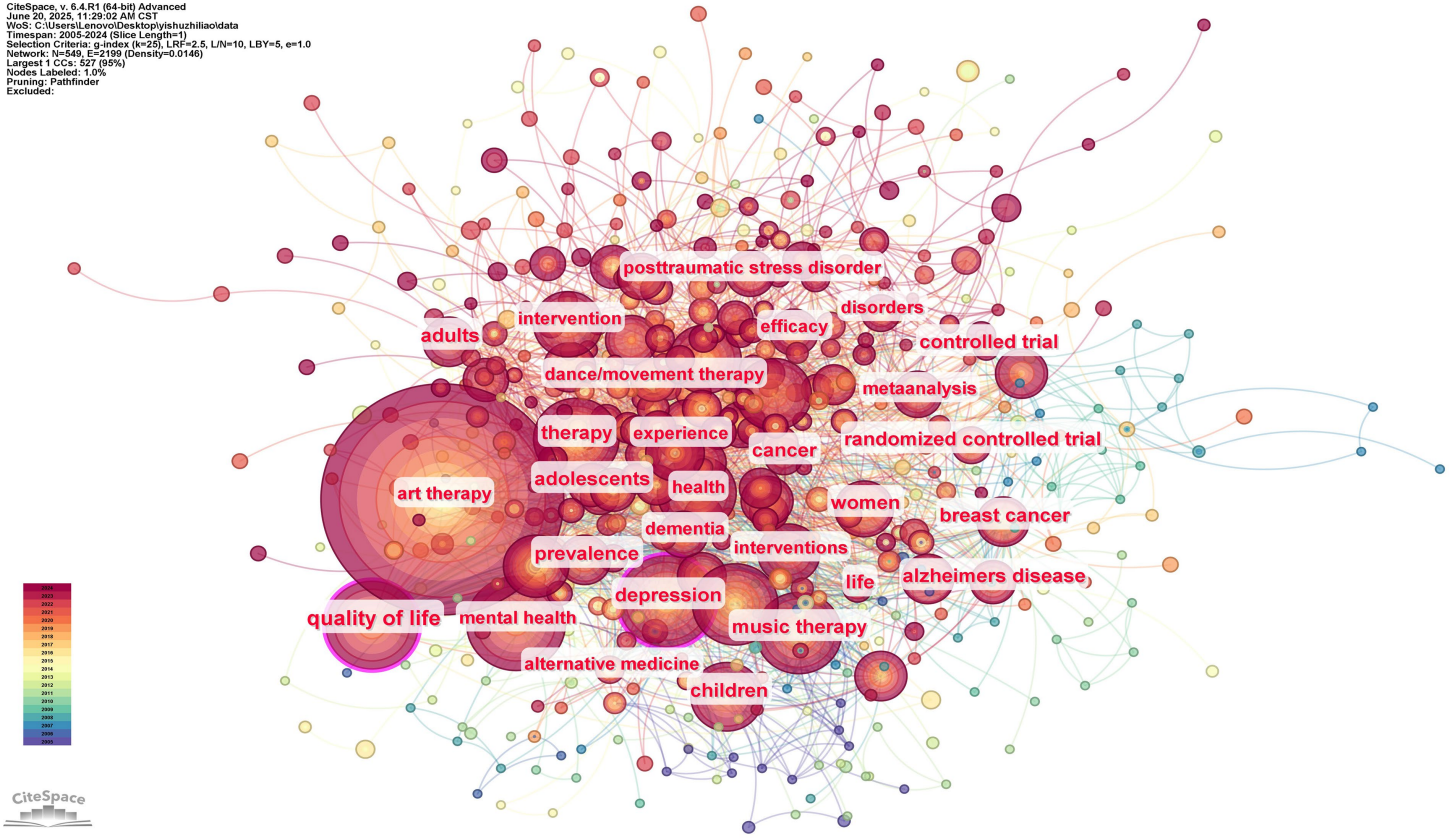
AT publications’ keyword clustering map, 2005–2024.

The keyword co-occurrence graph in Figure 8 was created by importing the data into CiteSpace software, choosing 2005–2024 as the time range, setting the time slice to one year, and representing the keywords with network nodes. The keyword appears more frequently the larger the node. According to the research, the grid density is 0.0146, and there are 549 nodes with 2,199 connections. The top ten rankings for keyword occurrence frequency in the AT domain from 2005 and 2024 are displayed in Table 8. The top six nodes were art therapy (757), quality of life (171), mental health (144), depression (136), music therapy (126), and therapy (125). The high-frequency keyword ‘art therapy’ serves as the core identifier for the field, anchoring the fundamental research scope of art therapy; keywords such as ‘quality of life’ and ‘mental health’ expand along paths that interpret practical values, define service recipients, and guide intervention directions, constructing a hierarchical progressive research framework that lays the foundational logic for knowledge production in the field. The correlation analysis of central indicators shows that art therapy is not an isolated theoretical field but a deeply embedded practice knowledge network within the mental health service system. Its theoretical linkage with public health issues such as ‘quality of life enhancement’ and ‘mental health improvement’, it forms an academic association mechanism across conceptual domains, highlighting its collaborative value in the ecological context of health services. In the dimension of disciplinary association, research presents an interdisciplinary paradigm of ‘medical demand-driven - art intervention practice - psychological efficacy verification’: Art therapy not only relies on medical evidence to confirm the efficacy in symptom improvement but also embeds within a psychological framework to achieve theoretical interpretation of the intervention mechanism; The coexistence of generalization and focus of ‘therapy’ further reveals its dual disciplinary dimensions that encompass both the universal norms of psychotherapy and the unique attributes of artistic media. In the trajectory of practical evolution, the focus of service has shifted from early childhood exploration to a refined study of “symptoms,” presenting an expansion trend toward coverage across all age groups and targeted symptom intervention. The intervention goals follow an advanced path from clinical treatment of diseases to health promotion, deeply echoing the paradigm shift in global health concepts from “disease cure” to “overall well-being enhancement.” This systemically highlights the diverse practical value of art therapy in health management systems and social welfare construction.

**Table 8:** Keyword co-occurrence list.

No.	Freq	Centrality	Year	Keywords
1	757	0.08	2005	art therapy
2	171	0.12	2006	quality of life
3	144	0.07	2009	mental health
4	136	0.10	2005	depression
5	126	0.05	2008	music therapy
6	125	0.06	2006	therapy
7	113	0.05	2007	health
8	108	0.03	2012	symptoms
9	104	0.08	2005	children
10	88	0.03	2006	anxiety

#### 3.3.2 Keywords co-occurrence time zone analysis

CiteSpace’s time-zone maps show research trends for each period and hotspots of change in the AT field to forecast future paths. The top five keywords for each of the four time periods into which the keywords in this analysis were divided are displayed in Table 9. From the perspective of academic evolution logic, from 2005 to 2009, ‘art therapy’ as a core identifier anchored the field foundation, with terms like ‘quality of life’ and ‘mental health’ outlining practical values and intervention directions, laying the initial framework for research; from 2010 to 2014, keywords such as ‘symptoms’ and ‘people’ emerged, reflecting the advancement of research toward symptom refinement and population expansion, focusing on the intervention of art therapy on different groups and specific symptoms; from 2015 to 2019, ‘trauma’ and ‘model’ focused on trauma intervention and theoretical model construction, embodying exploration of specific complex issues and disciplinary theorization; from 2020 to 2024, ‘dance therapy’ and ‘virtual reality’ highlighted the diversification of art forms and the integration of digital technology trends, catering to the needs of the times. Overall, the high-frequency keywords in each stage present an evolutionary trajectory from basic anchoring and practice expansion to in-depth exploration and innovative integration, reflecting the dynamic development and academic deepening of art therapy with social needs and technological advancements in research scope and methodological approaches.

**Table 9:** The top five high-frequency keywords for articles published on AT every 5 years from 2005 to 2024.

No.	Freq	Centrality	Year	Keywords
2005–2009				
1	757	0.08	2005	art therapy
2	171	0.12	2006	quality of life
3	144	0.07	2009	mental health
4	136	0.10	2005	depression
5	126	0.05	2008	music therapy
2010–2014				
1	108	0.03	2012	symptoms
2	86	0.04	2014	people
3	83	0.07	2010	women
4	55	0.03	2011	impact
5	50	0.02	2012	drama therapy
2015–2019				
1	40	0.03	2015	trauma
2	33	0.00	2018	model
3	30	0.01	2016	experiences
4	29	0.01	2015	pain
5	28	0.01	2016	management
2020–2024				
1	16	0.01	2020	dance therapy
2	13	0.00	2021	risk
3	11	0.00	2023	self
4	10	0.01	2020	benefits
5	9	0.00	2021	virtual reality

**Figure 9:**
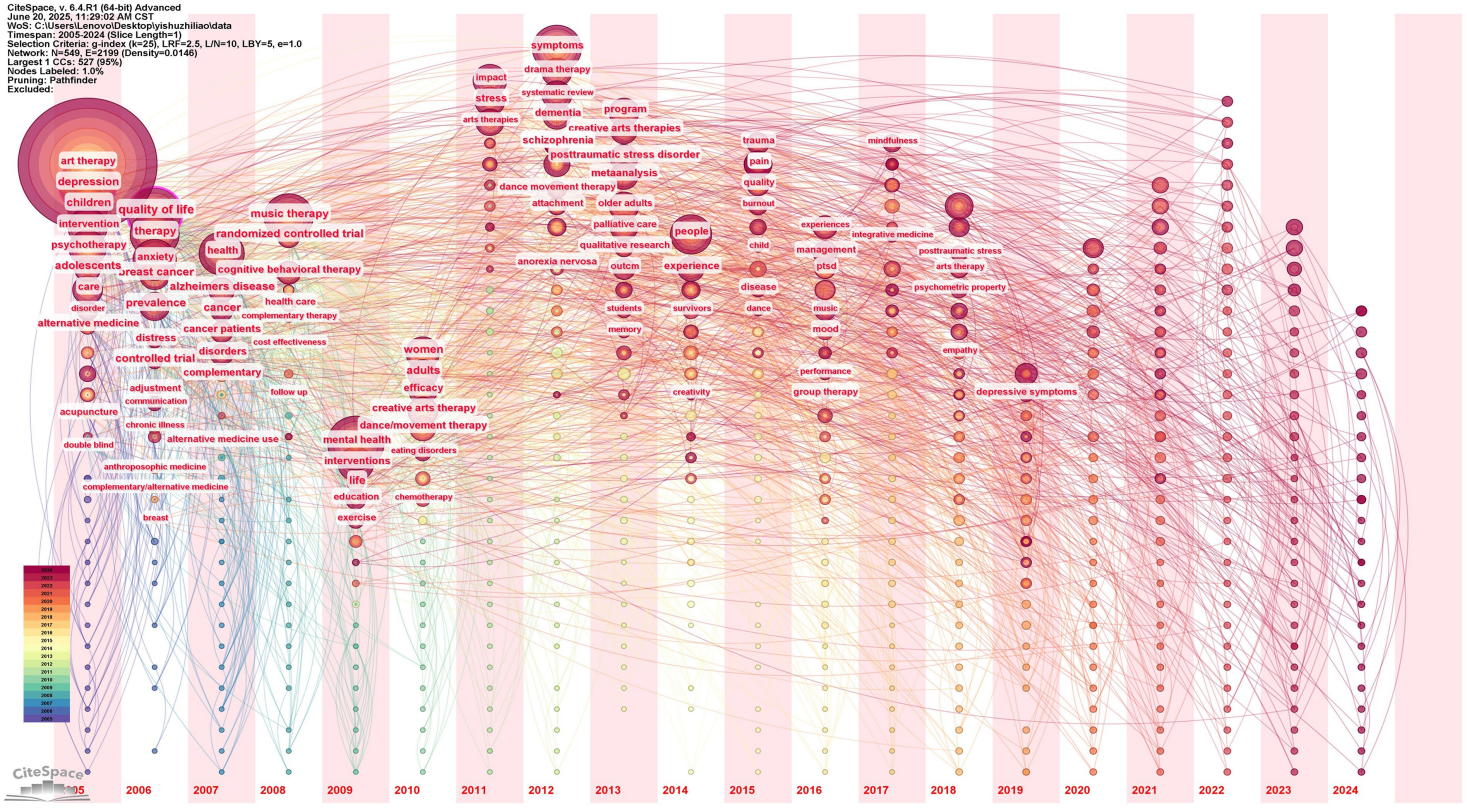
Annual changes in co-occurring keywords in AT papers, 2005–2024.

When the data was entered into CiteSpace, a timeline representation of the AT and AH studies from 2005 to 2024 was created (Figure 9). Examining high-frequency terms reveals a broad spectrum of studies in the AT domain, with distinctly different research priorities at different research stages. A map of the time zones in the AT domain (Figure 9) is shown below.

After a careful review of the pertinent literature, which includes high-frequency phrases from different years of AT study, the development of AT research has been broken down into the following three stages.

##### 3.3.2.1 Initial ground-breaking period (2005–2009)

The period from 2005 to 2009 is the initial foundation stage of art therapy research, with “art therapy” becoming the absolute core with ultra-high frequency, constructing the underlying logic of the field and clarifying “what art therapy is.”; As a typical segmentation form, “music therapy” verifies the healing logic from specific art media. At the same time, ‘mental health’ and ‘depression’ focus on intervention for common psychological issues, targeting basic clinical needs. ‘Quality of life’ extends to the social functional dimension of healing effects. Through the path of ‘defining core concepts → verifying subdivided forms → anchoring basic applications,’ a preliminary framework for field research is established, laying the foundation for the subsequent development of cognitive and practical cases.

##### 3.3.2.2 Expansion and deepening period (2010–2019)

This phase is further divided into two sub-phases. From 2010 to 2014, the research subjects and scenarios were expanded horizontally, with ‘people’ and ‘women’ reflecting an extension of the study to specific social identity groups, focusing on the adaptability of art therapy in diverse populations; ‘symptoms’ shifted the research from general psychological intervention to specific symptom improvement, emphasizing precision in outcomes; ‘impact’ focused on the multidimensional value of art therapy interventions; and ‘drama therapy’ enriched exploration of diverse art media, broadening the application boundaries. From 2015 to 2019, the intervention mechanisms and theoretical systems were deepened vertically, with ‘trauma’ and ‘pain’ targeting complex issues such as deep psychological trauma and comorbidities of mind and body, breaking through the boundaries of basic interventions; ‘model’ attempted mechanism modeling, abstracting practical experiences into theory; ‘experiences’ focused on qualitative research of individual healing processes, achieving a leap from ‘knowing it’s useful’ to ‘understanding why and how it can be more effective,’ solidifying the knowledge system.

##### 3.3.2.3 Innovation and integration period (2020–2024)

The period from 2020 to 2024 is characterized by an innovative integration phase, where art therapy embraces digital and interdisciplinary trends. The ‘virtual reality’ symbolizes the deep integration of ‘digital technology + art therapy,’ breaking through the traditional limitations of offline healing in terms of time and space and expanding the forms of intervention; ‘dance therapy’ continues to explore diverse artistic media. ‘Risk’ reflects a shift in research focus from single-minded attention to comprehensive assessment of intervention risks, pursuing safe and sustainable practices; ‘Self’ focuses on individual inner growth, uncovering the long-term psychological significance of healing, and ‘Benefits’ systematically summarizes multiple values, achieving a transformation from ‘traditional offline intervention’ to ‘digital empowerment and comprehensive evaluation,’ exploring new paths that adapt to future societal needs.

#### 3.3.3 The clustering analysis of keywords

**Figure 10:**
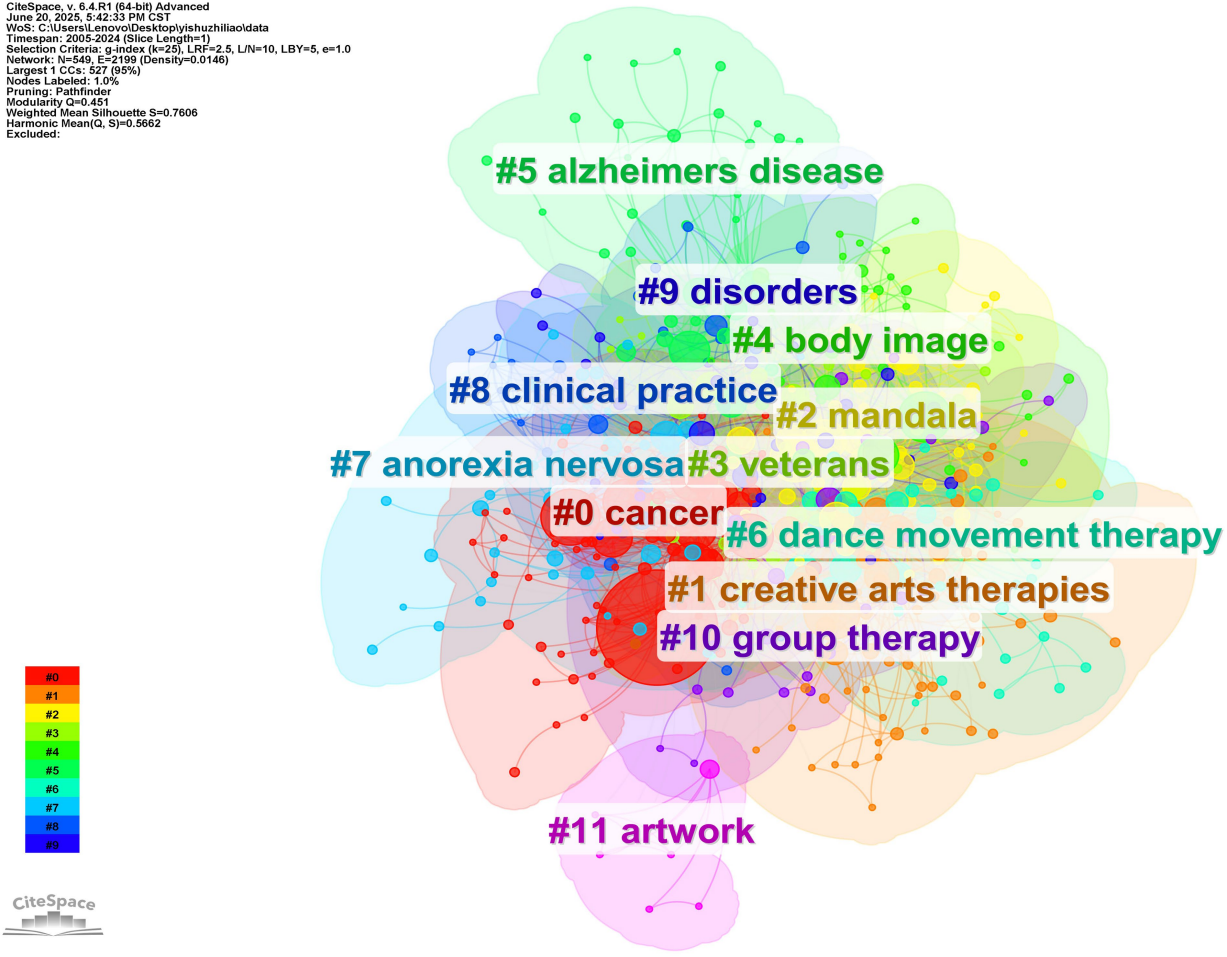
Clusters of AT papers and co-citation networks, 2005–2024.

This paper analyzes the knowledge graph for keyword clustering and exported using CiteSpace (Figure 10). The evolution of AT throughout time and research themes are examined. The chosen time frame is 2005–2024, and Figure 10 shows that we have twelve automatically formed clusters:#0 cancer;#1 creative arts therapies;#2 mandala;#3 veterans;#4 body image;#5 alzheimers disease;#6 dance movement therapy;#7 anorexia nervosa;#8 clinical practice;#9 disorders;#10 group therapy;#11 artwork.

CiteSpace provides modularity and silhouette metrics based on the clarity of the network structure and clustering, i.e., modularity (Q-value) and average silhouette (S-value) metrics, which can be used as a basis for us to judge the effectiveness of the mapping. In general, the Q value is usually between 0 and 1; when the value is greater than 0.3, it means that the structure of the delineated associations is significant; when the S value is 0.7, the study is usually regarded as persuasive, and a value close to 1 indicates that the consistency of the articles in the clustering is high (Alaeddini et al., 2023). The Q value of 0.451 in Figure 10 indicates that the structure of the associations delineated by the study is significant; the S value of 0.7606 indicates that the clustering is persuasive. There are three methods for class clustering calculation: LLR, LSI, and MI. Through the practice of the article, we found that LLR is more practical and less repetitive, so we chose LLR as the primary calculation method (Tang et al., 2022).

In #0 - #11, the smaller the number, the more keywords are included in the set. The largest cluster (#0) in Table 10 contains 92 articles with a silhouette value of 0.81, which is greater than 0.7, indicating that the total core consistency of the articles in this cluster is high. The studies mainly focused on CANCER, QUALITY OF LIFE, ART THERAPY, BREAST CANCER, ONCOLOGY, etc.; most were published in 2009. The second largest category (#1) contains 73 articles on creative arts therapies, drama therapy, music therapy, etc. The silhouette value of #1 is 0.706, and most of its articles were published in 2014. The third major category (#2) contains 65 papers with a silhouette value of 0.644, which is less than 0.7, indicating that the total core consistency of the articles in this cluster is not high, where the main content is a mandala, health care, poetry, and mental health services, among others. Nine clusters are derived from CiteSpace, and Table 10 details the main elements in each of the nine clusters.

**Table 10:** List of AT-related records and cited clusters from 2005–2024.

Cluster ID	Size	Silhouette	Mean (Year)	Label (LLR)
0	92	0.81	2009	cancer (74.75, 1.0E-4); quality of life (41.87, 1.0E-4); art therapy (38.89, 1.0E-4); breast cancer (38.81, 1.0E-4); oncology (29.46, 1.0E-4)
1	73	0.706	2014	creative arts therapies (43.7, 1.0E-4); arts therapies (39.17, 1.0E-4); drama therapy (39.01, 1.0E-4); creative arts therapy (33.33, 1.0E-4); music therapy (20.18, 1.0E-4)
2	65	0.644	2014	mandala (8.47, 0.005); health care (8.47, 0.005); art therapist (8.47, 0.005); poetry (8.47, 0.005); mental health services (8.47, 0.005)
3	45	0.762	2017	veterans (34.41, 1.0E-4); ptsd (30.52, 1.0E-4); traumatic brain injury (15.38, 1.0E-4); military (15.2, 1.0E-4); memory reconsolidation (15.16, 1.0E-4)
4	41	0.73	2014	body image (14.06, 0.001); group art therapy (14.06, 0.001); computer system (9.62, 0.005); psychometric property (9.62, 0.005); sexual abuse (9.62, 0.005)
5	39	0.855	2012	alzheimers disease (24.31, 1.0E-4); dementia (23.03, 1.0E-4); systematic review (23.01, 1.0E-4); mild cognitive impairment (13.39, 0.001); group psychotherapy (10.73, 0.005)
6	36	0.722	2017	dance movement therapy (15.14, 1.0E-4); cancer (12.07, 0.001); randomized controlled trial (10.67, 0.005); intellectual disability (9.59, 0.005); arts therapy (7.62, 0.01)
7	35	0.783	2018	anorexia nervosa (33.1, 1.0E-4); eating disorders (28.41, 1.0E-4); virtual reality (27.55, 1.0E-4); neurorehabilitation (18.13, 1.0E-4); mixed methods (15.7, 1.0E-4)
8	33	0.775	2014	clinical practice (20.44, 1.0E-4); therapeutic process (13.61, 0.001); uk (13.61, 0.001); survey (9.86, 0.005); controlled trial (7.09, 0.01)
9	31	0.774	2018	disorders (18.71, 1.0E-4); trial (12.46, 0.001); predictors (12.46, 0.001); predictive processing (12.46, 0.001); motor (12.46, 0.001)
10	30	0.851	2018	group therapy (26.58, 1.0E-4); palliative care (15.04, 0.001); healthcare providers (12.73, 0.001); child sexual abuse (12.73, 0.001); death education (12.73, 0.001)
11	7	0.991	2007	artwork (10.2, 0.005); art norms (10.2, 0.005); seizure (10.2, 0.005); art therapy: assessment (10.2, 0.005); neurobiology (10.2, 0.005)

Four of the twelve clusters were founded in 2014, suggesting that the AT field saw a surge in that year with a wide range of research and relatively strong interdisciplinary cross-collaboration. In addition, ‘virtual reality’ in #7 Anorexia Nervosa has formed a co-occurrence network with keywords such as ‘eating disorders’ and ‘neurorehabilitation,’ proving that virtual reality technology plays an important role in the intervention of anorexia nervosa combined with neurorehabilitation as an emerging technology. The hot changes and upcoming trends in the AT field development are evident when the keyword clustering and co-occurrence mentioned above are combined.

#### 3.3.4 Research clustering timeline

**Figure 11:**
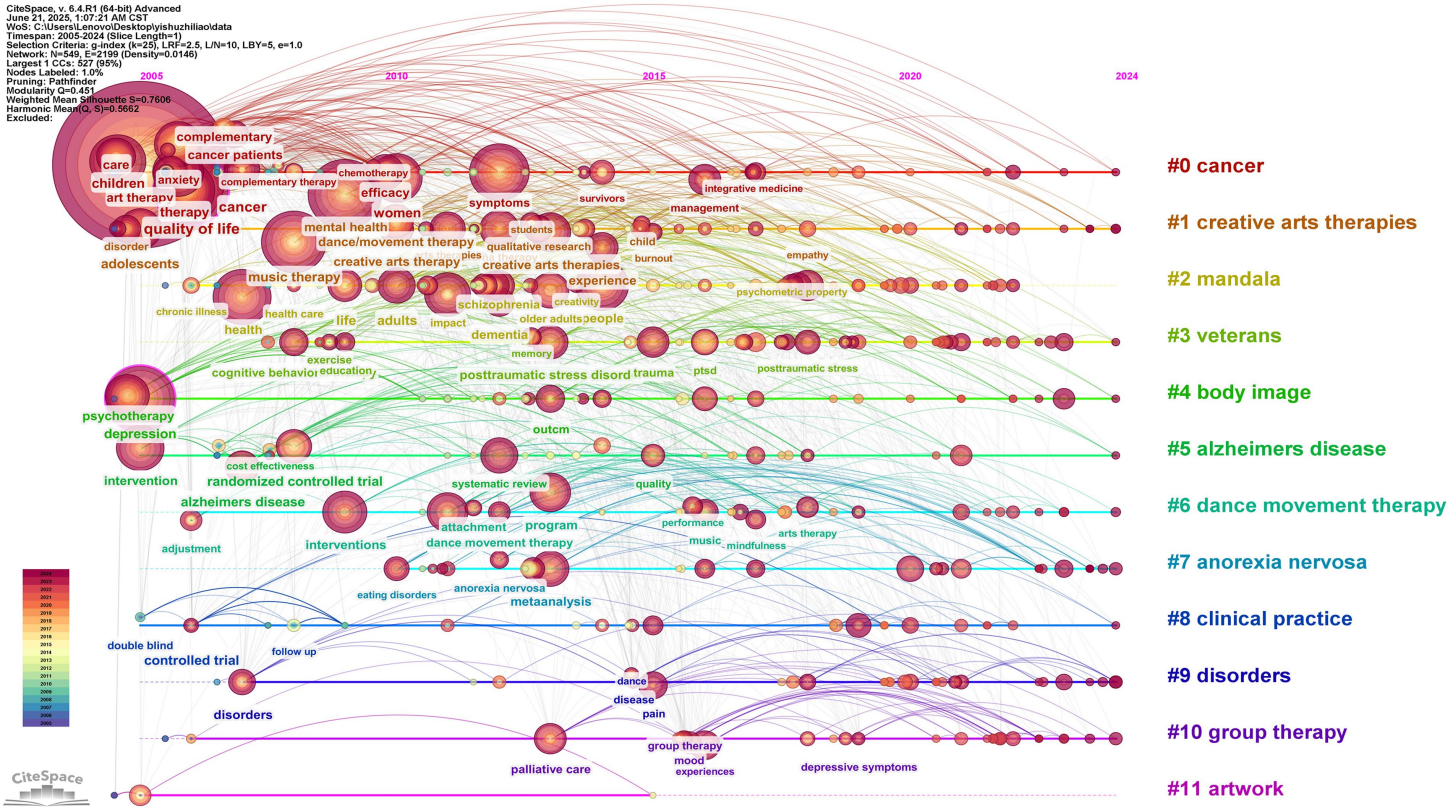
Timeline chart for the 2005–2024 study.

CiteSpace clustering timeline map can reveal the research dynamics of high-frequency keywords within each cluster over time. This helps to systematically analyze the emergence, flourishing, and decline of research hotspots in the AT field in different periods and realize the fine analysis of the research focus in a specific period through the precise division of time sequence. The specific information is shown in Figure 11.

Based on the timeline generated from keywords in CiteSpace, these 12 clusters can be categorized into five core themes.

1. Focusing on the application of art healing interventions for patients with various diseases.
2. Focusing on the specific implementation of art therapy.
3. Involving the phenomenon of burnout and the topic of mental health.
4. Art healing for people with different characteristics.
5. About the practice scenarios and effect evaluation of art healing.

**Figure 12:**
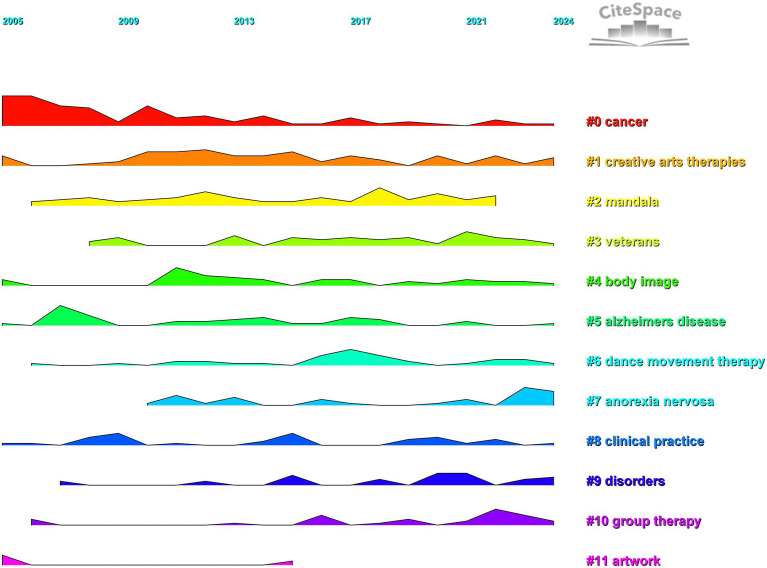
Land space map of the AT study, 2005–2024.

CiteSpace generates clustered timeline maps and land space maps of art healing research, which more intuitively present the development trajectory and heat change of each research topic in art healing. In the cross-correlation between the horizontal time axis and the vertical clusters in the cluster land space map, different color curves correspond to specific research clusters (e.g., #0 cancer, #1 creative arts therapies, etc.), and their undulation patterns carry information about the dynamic evolution of research intensity. In terms of development, the basic conceptual clusters represented by #1 creative arts therapies appeared earlier, forming the cornerstone of the research; with the advancement of the research, subclusters targeting specific diseases (#0 cancer, #5 alzheimers disease), specific groups (#3 veterans), and specific forms of healing were derived (#6 dance movement therapy), mapping the deepening of the research from broad conceptual interpretation to precise application and diversified scenarios. Figure 12 presents additional specific information.

Focusing on the trend of heat, clusters such as #1 creative arts therapies and #0 cancer have maintained fluctuating heat for a long time and become “evergreen themes” in the field of art healing, which is driven by the continuous exploration of mechanism and optimization of practice; some clusters (such as #2 mandala, #11 Some clusters) (e.g., #2 mandala, #11 artwork) can be regarded as emerging potential directions of art media application if their curves show an upward trend in the later stage. On the contrary, declining clusters may be dormant due to bottlenecks or shifts in the research direction. Overall, the chronological distribution map supports sorting out the development of the art healing field, identifying the core and potential research directions, and helping to anchor the research value and trend evolution logic accurately.

#### 3.3.5 Analysis of research trends

“Mutations” are words that undergo significant alteration and are frequently used in literature in a brief amount of time. They can determine what is trendy in a field of study.

**Figure 13:**
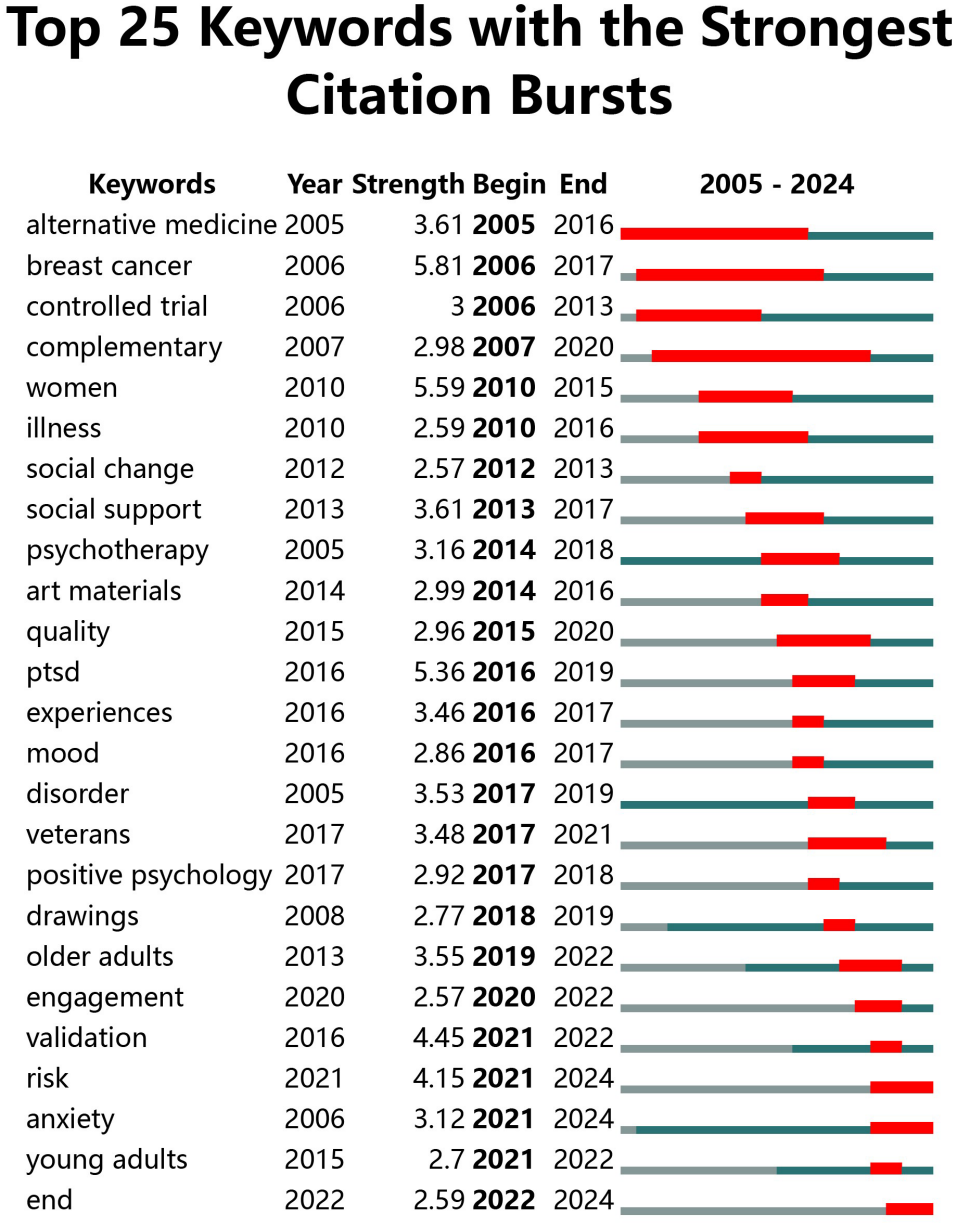
Hotspot emergent words for AT.

Using CiteSpace’s burst word recognition approach, 25 burst words were found between 2005 and 2024 (Figure 13). As can be seen from Figure 13, Research areas and hotspots in different stages differ significantly from one another. This further supports the evolutionary trajectory of the previously indicated study hotspots. From the figure, it can be seen that from 2005 to 2010, the terms “alternative medicine” and “psychotherapy” started the research prelude, focusing on integrating alternative medicine and clinical verification of diseases. From 2010 to 2020, terms such as “women” and “art materials” expanded the research objects and scenarios and deepened the dimension of medium and efficacy. Beyond 2020, terms such as “veterans” and “positive psychology” drive the field toward group-specific, interdisciplinary integration and refined assessment. At the same time, high-intensity, long-run themes such as “breast cancer” and ongoing expansion themes such as “alternative medicine” have been the most popular. It highlights the interdisciplinary character and the evolutionary logic from “validity exploration” to “precise scientific station.” It provides support for sorting out the veins of the field and anchoring the points of innovation. AT research will focus on the following four areas in the coming period by analyzing the emerging keywords.

1. Specific group interventions: Centered around veterans, older adults, and young adults.” In-depth exploration of the precise intervention mode of art healing for different age and identity groups, such as healing programs for veterans with PTSD and the elderly with cognitive decline.
2. Healing mechanism and assessment: Through “validation” and “controlled trial,” the scientific validation of art healing is strengthened, and the mechanism of action is clarified (e.g., how the art materials and forms affect). (e.g., how art materials and forms affect psychology), Moreover, a standardized healing assessment system should be built.
3. Interdisciplinary fusion practice: Continuing the association of “positive psychology” and “psychotherapy,” promoting the intersection of art healing with psychology and medicine, expanding the application to psychological problems such as “anxiety” and “disorder,” and improving the intervention pathway.
4. Emerging scenarios and forms: Relying on “engagement” and “art materials,” we will explore the potential of new media, such as mandalas and digital art, and expand the practice scenarios of community and recreational institutions—innovative forms of healing activities (e.g., group art creation, online healing).

## 4 Discussion

**Figure 14:**
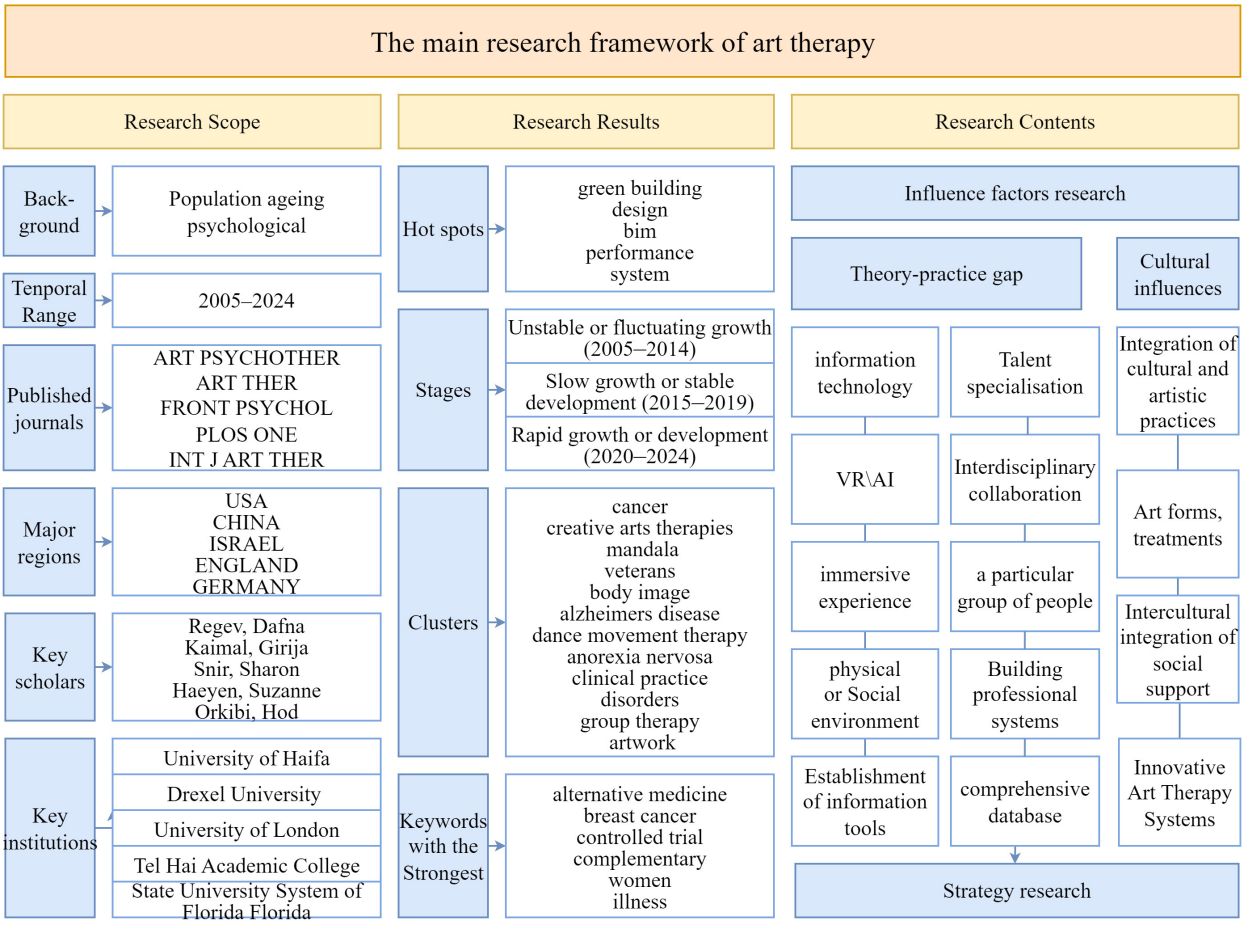
Mainstream frameworks in AT research.

This study analyses Web of Science core data on art therapy (AT) and art healing (AH) to explore the evolution of research on the integration of art and psychotherapy, thus tracing the direction of research themes and temporal dimensions within this interdisciplinary field. First, a systematic compilation of relevant data was used to lay the foundation for subsequent analyses. Subsequently, we examine multiple dimensions to forecast the field’s future development, including the types and scopes of relevant journals, the number of publications, the collaboration patterns among authors, and the geographical distribution of research areas and their affiliated institutions. The details are shown in Figure 14. By incorporating keyword co-occurrence maps over time, it is possible to identify changes in research hotspots during the AT’s development (Liu et al., 2021). This field has a low density of interdisciplinary collaboration and dispersed, cross-cutting research keywords. Research using keyword clustering can create a scientific categorization, pinpoint the area’s research hotspots, and provide recommendations for more studies.

To infer future research directions for art therapy (AT), we analyzed keywords including “older adults,” “young adults,” “controlled trial,” “positive psychology,” and “art materials.” These keywords guide us to explore specific areas deeper: developing group-tailored interventions, deciphering healing mechanisms and assessment methods, promoting interdisciplinary integration in practice, and exploring emerging application scenarios and forms. Beyond these keyword-driven directions, the broader societal context further underscores the urgency of AT research. Notably, the COVID-19 pandemic has imposed profound impacts on the global population’s mental health, while the aging of the world’s population exacerbates the mental health burden on older adults (Kola et al., 2021; Reynolds et al., 2022). In this context, AT emerges as a critical tool to address these challenges, creating an imperative to develop optimized AT systems and advance the field’s evolution.

From the data analysis in the article, the development of art healing has multiple shortcomings. In terms of cross-cultural research and geographical development, the West dominates Western research, non-Western theories and practices are missing, the international cooperation network of Asian institutions is fragmented, and cultural differences also affect the universality of theories in terms of the combination of technological applications and theories, the empirical basis of emerging technologies, such as virtual reality, is weak, and theory and practice are disconnected, with the absence of practice guidelines; in terms of research on special groups and scenarios, there is insufficient coverage of specific populations, and the application of community and educational scenarios is inadequate. In terms of interdisciplinary research and methodology, there is insufficient interdisciplinary integration, imbalance between quantitative and qualitative research, data lag, and sample bias; in terms of ethical and assessment systems, there is insufficient discussion of ethical risks and a single dimension of efficacy assessment.

Future research should prioritize the expansion of art therapy (AT) applications. While the integration of art and psychotherapy has shown positive results in areas such as emotion regulation and personality disorder treatment, its expansion to more diverse populations and settings remains an area for continued exploration. For example, individualized art therapy programs can be tailored to specific populations and art therapy practices that enhance cross-cultural research. Precision intervention for special groups such as veterans and the elderly and constructing a three-tier intervention model of “group portrait - media matching - effect tracking” (e.g., mandala painting combined with cognitive training for dementia patients) have more evidence-based medical value than traditional programs. There are differences in how people understand and express art in different cultures, and art therapy can be better promoted globally through cross-cultural research. Secondly, a more systematic art therapy theory and method system should be established. There are many different types of art therapy, including dance, drama, music, sound, and visual arts therapy, but the integration and synergy between these therapies require further research and practice. Next, in terms of technology application, the integration of virtual reality (VR), artificial intelligence (AI), and other emerging technologies with art therapy is worth looking forward to in order to construct a “digital art therapy ecosystem.” The combination of VR immersion experience and fMRI brain area monitoring can realize the precise quantification of the intervention effect. This breaks through traditional art therapy’s time and space limitations and provides an innovative technological path for mental health services; AI technology can assist in analyzing the patient’s artwork, mining the psychological information contained therein, and providing a more accurate basis for formulating treatment plans.

For the sake of this essay, AT and AH share a novel paradigm that encourages the fusion of psychology and art. This paradigm relies on developing alternative medicine as an effective model for addressing psychological issues and improving quality of life. This paper aims to prevent AT from being evaluated in isolation from other creative qualities and promote the development of various AT performance evaluation parameters. The theoretical contribution of this study lies in four main areas: (1) The time frame of this study is long, and the focus of the study is realistic and socially progressive; (2) This study proposes that art therapy is affected by the art industry, the art atmosphere, and the professionals, and adopts a quantitative research method to systematically study the art class, the art industry, and art therapy, in order to offer new insights on the evolution of AT theory; (3) Drawing from prior studies, the development of the subjective AT theory (Subjective art therapy theory refers to a theoretical framework that emphasizes an individual’s subjective experience and interpretation in art therapy, in contrast to objective, evidence-based models. It emphasizes the therapist’s intuitive involvement in the client’s artistic expression to facilitate emotional healing.); (4) The time zone map in CiteSpace can automatically display the clustering scale and progression of research topics in the time dimension, but it cannot comprehensively display each research stage’s contents. Through the co-occurrence history and co-occurrence record of the AT subject literature, we consider the publication time, quantity of downloads, and citations in this paper. Provides a more thorough overview of the history of AT research and include both macro and micro viewpoints in future studies and sustainable development initiatives.

This study has some limitations at the methodological level. The study is supported only by the WoS core database, which is comprehensively indexed and CiteSpace-adapted. However, this has resulted in insufficient coverage of non-English-speaking countries, such as China’s “ink therapy” and Japan’s “aesthetic therapy of object sorrow.” For example, Chinese “ink therapy” and Japanese “aesthetic therapy of object sorrow” and other localized practices account for less than 5% of the data. Meanwhile, Scopus, as an abstract and citation database with broader coverage, has decreased compatibility with CiteSpace due to the platform update in 2017, while PubMed exported data is limited by missing citation information, and both are difficult to satisfy the analytical needs of CiteSpace, which limits the breadth of data coverage of the present study to a certain extent (Singh et al., 2021). In addition, although the keyword search strategy can eliminate interfering items, it is challenging to avoid missing cross-disciplinary studies such as “art therapy in HIV psychological interventions,” there is a blind spot in capturing emerging terms such as “VR art therapy.” The quantitative analysis method of CiteSpace cannot explore the deep mechanism of trauma expression through art creation in the qualitative study, which needs to be supplemented with interviews and other qualitative research methods.

At the data level, the study is equally problematic. On the one hand, there is a lag in the inclusion of post-2023 literature, which prevents the inclusion of the latest applications, such as virtual reality technology, and makes it challenging to present a comprehensive picture of the latest developments in the field. On the other hand, the regional distribution of data is imbalanced. Research from countries such as the United States and the United Kingdom accounts for over 40%. In contrast, theoretical constructions of non-Western models such as ‘drum therapy’ in Africa and ‘ritual healing’ in Southeast Asia have not yet been systematized, showing a clear bias toward ‘Western centrism.’ Moreover, there is a high proportion of clinical intervention studies and a relative lack of applied research in community and educational settings (e.g., practice in Hong Kong, China), highlighting the geographical imbalance in “theory-practice” translation.

The theoretical and ethical dimensions also reveal the limitations of the study. The current theoretical framework is centered on Western psychology, with insufficient integration of Eastern theories such as “mind–body unity”; for example, the Japanese “body narrative therapy” was not included in the cluster analysis, affecting the theory’s comprehensiveness and universality. In addition, ethical risks have not been adequately explored in the research, and issues such as lay practitioners’ handling of patient trauma disclosures and privacy protection in digital therapy have not been discussed in depth. These potential risks pose a threat to the safety of the practice of art therapy.

Based on the limitations of existing research in terms of methodology, data, and theoretical ethics, future art therapy research can be explored in multiple dimensions: in terms of cross-cultural integration, through the joint search of multiple databases such as WoS, CNKI, Scopus, etc., we can deeply explore localized modes such as “Positive Thinking Painting Therapy” in China and “Drumming Therapy” in Africa.” Regarding research methodology, CiteSpace quantitative analysis combines qualitative methods such as IPA and rootedness theory to analyze art therapy’s emotional mechanisms and pathways comprehensively. In terms of research methodology, CiteSpace quantitative analysis is combined with qualitative methods such as IPA and rootedness theory to achieve a comprehensive analysis of art therapy’s affective mechanisms and pathways; in the field of technological ethics and fairness, we will explore low-cost alternatives to VR, AI, and other technologies such as mobile phone APP therapeutic tools in resource-poor regions, and establish a sound ethical code to protect privacy and practice safety; for special groups and emerging scenarios, we will strengthen precise interventions for the elderly in the case of cognitive decline, veterans in case of PTSD, etc., and incorporate these into their physiology. For special groups and emerging scenarios, we will strengthen precise interventions for special groups, such as the elderly with cognitive decline and veterans with PTSD, and design personalized programs based on their physiological and psychological characteristics. The advancement of the above research directions is expected to improve the theory and practice system of art therapy and further promote its development and application in the modern medical field.

## 5 Conclusion

Based on the core database of Web of Science and the CiteSpace visualization tool, this study systematically analyses 1,799 kinds of literature in art therapy (AT) from 2005 to 2024 to reveal its research lineage, hotspot distribution, and future direction. The study found that significant progress has been made in AT’s theoretical construction and practical application. However, structural limitations remain, and interdisciplinary innovation and global collaboration are needed to break through the existing bottlenecks.

In terms of development stages, AT research has shown a “stepped growth” in the past 20 years, with research going through three phases: the embryonic start-up period, the steady growth period, and the rapid rise period. The budding stage (2005—2014) saw an annual publication volume of less than 70 articles, with research focusing on defining basic concepts and preliminary clinical validation. This was limited by theoretical fragmentation and barriers between disciplines; during the stable growth period, the annual publication volume increased to 134 articles, driven by mental health crises and the popularization of technology, which propelled research toward symptom segmentation and population expansion; in the stable growth period (2015—2019), the annual publication volume further increased to 134 articles, continuing the trend of research extension into symptom segmentation and population coverage; entering the rapid rise phase (2020—2024), the peak annual publication volume reached 222 articles, with COVID-19 psychological intervention needs and interdisciplinary technologies such as ‘Neuroart Therapy’ becoming core driving forces, and emerging directions like virtual reality and dance therapy experiencing explosive growth. In terms of discipline ecology and institutional characteristics, the United States, the United Kingdom, and Israel contributed 63% of the core literature, and although China ranked second in terms of the number of publications (177), clinical interventions were the primary focus, and there was a lack of local theoretical construction; European and American institutions, such as the University of Haifa (107) and Drexel University (46), dominated the research, but the density of the cross-institutional co-operation network was only 0.0047. The Asian institutions did not have enough international collaborations. In terms of research hotspots, there has been a shift from ‘single disease intervention’ to ‘technological integration and whole-life care’: The basic research phase (2005—2014) focused on ‘mental health,’ ‘depression,’ and ‘music therapy’ as core topics, with an emphasis on effectiveness verification; the application expansion phase (2015—2019) highlighted keywords such as ‘trauma,’ ‘models,’ and ‘experience,’ moving toward complex psychological interventions and theoretical mechanism modeling; the innovative integration phase (2020—2024) featured ‘virtual reality,’ ‘risk,’ and ‘self’ at the forefront, reflecting the need for digital technology embedding and ethical assessment system construction.

Based on the current research, AT is an expanding field of study. The research hotspots of AT are increasing year by year. The breadth of research areas has expanded, and there is a growing body of literature about AT across disciplines. The journals are relatively widely distributed, spanning a variety of disciplines. Publication collaboration varies by nation, with some groups exhibiting less cooperation than others. Nonetheless, the tight ties between academics and publishers, as well as between some of the scholars themselves, demonstrate that, in the context of globalization, close collaboration between many nations, publishers, and institutions can push a discipline forward. According to this report, institutions in the field of AT research do not cooperate. They do not cooperate with others and are primarily internally centered. The keywords used in this study demonstrate that AT includes AH, which is an important pillar in the development of AT. Future AT research should concentrate on PTSD recovery, virtual reality, mental health, and geriatric care. In our view, the following areas should be focused on in future developments:

1. The discrepancy between practice and theory.
2. Cross-cultural integration.
3. Creation of multiple dimensions of quality and impact assessment.
4. Virtual reality interventions and development in AT.

This study systematically analyzes the cutting-edge theoretical and practical landscape of the art therapy (AT) field, revealing the core challenges and transformation paths it faces in its interdisciplinary development. The study points out that art therapy needs to break through the traps of “techno-utopia” and the cultural bias of “Western centrism” and promote the transition of theoretical construction from a single dominant paradigm to the paradigm of “global dialogue.” Specifically, researchers need to strengthen interdisciplinary collaboration with ethicists and technology developers to build a research framework that takes into account both technological innovation and humanistic care; clinicians should enhance cultural sensitivity, incorporate Indigenous art forms (e.g., the East Asian view of “mind–body unity” and African drumming therapies) into their practice, and strictly follow ethical norms to safeguard clinicians should enhance cultural sensitivity, incorporate indigenous art forms (such as the East Asian concept of “unity of mind and body” and African drum therapy) into their practice, and strictly follow ethical norms to ensure the safety and compliance of the treatment process.

Future research should be social justice orientated, focusing on key areas such as therapeutic talent training, innovation in artistic practice, sustainable development models, intervention for special groups, integration of virtual reality technology, and construction of multi-dimensional assessment systems in order to systematically address global issues such as mental health crises, social isolation, and cultural differences. By building an inclusive theoretical model and practical framework, art therapy is expected to provide innovative solutions for the modern mental health system, promote the deep integration of art, psychology, and medicine, and ultimately realize the comprehensive development of the field in equity and sustainability. This study provides AT researchers, clinicians, and related practitioners with theoretical depth and practical orientation, helping them grasp the discipline’s trend accurately and carry out cutting-edge exploration.

## Data Availability

The original contributions presented in the study are included in the article/supplementary material, further inquiries can be directed to the corresponding author.
